# Broad Metabolome Alterations Associated with the Intake of Oral Contraceptives Are Mediated by Cortisol in Premenopausal Women

**DOI:** 10.3390/metabo11040193

**Published:** 2021-03-24

**Authors:** Clara Eick, Johanna Klinger-König, Stephanie Zylla, Anke Hannemann, Kathrin Budde, Ann Kristin Henning, Maik Pietzner, Matthias Nauck, Henry Völzke, Hans J. Grabe, Johannes Hertel

**Affiliations:** 1 Department of Psychiatry and Psychotherapy, University Medicine Greifswald, D-17489 Greifswald, Germany; clara.eick94@gmail.com (C.E.); hans.grabe@med.uni-greifswald.de (H.J.G.); or johannes.hertel@nuigalway.ie (J.H.); 2 Institute of Clinical Chemistry and Laboratory Medicine, University Medicine Greifswald, D-17489 Greifswald, Germany; stephanie.zylla@med.uni-greifswald.de (S.Z.); anke.hannemann@uni-greifswald.de (A.H.); kathrin.budde@med.uni-greifswald.de (K.B.); ann-kristin.henning@med.uni-greifswald.de (A.K.H.); maik.pietzner@charite.de (M.P.); matthias.nauck@med.uni-greifswald.de (M.N.); 3 German Centre for Cardiovascular Research (DZHK), Partner Site Greifswald, D-17489 Greifswald, Germany; voelzke@uni-greifswald.de; 4 Institute for Community Medicine, University Medicine Greifswald, D-17489 Greifswald, Germany; 5 German Center for Neurodegenerative Disease (DZNE), Site Rostock/Greifswald, D-17489 Greifswald, Germany; 6 School of Medicine, National University of Ireland, H91 CF50 Galway, Ireland

**Keywords:** oral contraceptives, metabolome, cortisol, mediation

## Abstract

The use of oral contraceptives (OCs) has been associated with elevated blood cortisol concentrations. However, metabolic downstream effects of OC intake are not well described. Here, we aimed to determine if the blood metabolome is associated with the use of OCs and to estimate if these associations might be statistically mediated by serum cortisol concentrations. Plasma metabolites measured with the Biocrates Absolute*IDQ* p180 Kit and serum cortisol concentrations measured by an immunoassay were determined in 391 premenopausal women (116 OC users) participating in two independent cohorts of the Study of Health in Pomerania (SHIP). After correction for multiple testing, 27 metabolites were significantly associated with OC intake in SHIP-TREND (discovery cohort), of which 25 replicated in SHIP-2. Inter alia, associated metabolites included 12 out of 38 phosphatidylcholines with diacyl residue, 7 out of 14 lysophosphatidylcholines and 5 out of 21 amino acids. The associations with phosphatidylcholines were statistically mediated by cortisol, whereas lysophosphatidylcholines showed no mediation effect. The results represent a step toward a better understanding of the metabolic consequences of OC intake. Connecting cortisol with metabolic consequences of OC intake could help to understand the mechanisms underlying adverse effects. The blood metabolome may serve as a biomarker for identifying users at high risk for developing such adverse effects.

## 1. Introduction

Oral contraceptives (OCs) are on the list of essential medicines and, all over the world, are frequently used to prevent pregnancy [1,2]. Nevertheless, numerous side effects of OC intake have been described [3], including alterations of triglyceride and lipoprotein concentrations [4], increased risk of venous thrombosis [5] by modulating procoagulant and fibrinolytic factors [6,7], reduced insulin sensitivity [8], elevated cortisol concentrations [9,10,11] and a higher risk of depression [12].

The physiological menstrual cycle in women not using OC is determined by periodic hormonal changes, such as varying estrogen concentrations. OCs consist of different compositions of sex hormones to attenuate hormonal changes during the menstrual cycle. Importantly, hormonal concentrations impact metabolism. In rodents, estrogen, for example, was associated with lipid and glucose homeostasis, including inhibition of lipolysis and an improvement of insulin sensitivity [13,14,15]. Hence, it is plausible that the use of OCs has consequences for the metabolic equilibria of the female body. Especially since OCs are frequently used over several years, the metabolic consequences are a relevant research target in understanding the risk of adverse effects.

One way to analyze these impacts of OCs is via analyses of omics data generated by high-throughput methods, providing holistic information on a biological system [16,17]. Metabolomics is focusing on low molecular weight biological molecules coming from the human superorganism, including the microbiome [18]. Such holistic metabolite measures in human body fluids obtain information on metabolic activity by measuring the products and substrates of biochemical pathways reflecting cellular metabolism and human physiology [17]. Besides static traits such as the genetic background of the organism, the metabolome is heavily influenced by dynamic factors such as nutrition [19], exercise [20], coffee consumption [21], nicotine [22], drugs [23] and the menstrual cycle [24,25].

Cortisol, a steroid hormone secreted in a pulsatile pattern with a circadian rhythm, was observed to be drastically elevated in women taking OCs [9,10,11]. Additionally, cortisol concentrations rise in situations of acute and chronic stress, but the beneficial effects of this stress reaction are reversed if cortisol concentrations remain upregulated over a longer time [26]. Particularly within the context of the stress response, cortisol has been described as a major regulator of gluconeogenesis [27]. Moreover, studies in rodents demonstrated inhibition of lipogenesis [28]. Thus, elevated cortisol concentrations were linked to insulin resistance and visceral adiposity, as well as psychological disorders such as depression and anxiety [29,30]. Nevertheless, potentially mediating biological mechanisms are still elusive.

Here, our main goal was to test the hypothetical association between OC intake and altered plasma metabolite concentrations. Secondly, we investigated whether the observed OC-associated patterns in the blood metabolome were statistically mediated by serum cortisol concentrations, delivering insight on cortisol-related metabolome changes. All analyses were based on two independent population-based samples.

## 2. Results

Data of 391 premenopausal women were analyzed (SHIP-TREND: n = 232, SHIP-2: n = 159). Separate descriptive statistics for both cohorts can be found in Table 1. OC intake was reported by 73 women in SHIP-TREND and 43 women in SHIP-2, respectively. OCs consist of either a single compound, which is progesterone, or they consist of combined compounds to which estrogen is added. Whereas the use of combined compounds is paused after an intake of 21 days for 6 to 7 days, the use of the progesterone-only pill is continued. The majority of women in SHIP-TREND (95.9%) and SHIP-2 (97.7%) used products with combined compounds (Tables S1 and S2). Hence, data was not suitable to test the impact of single vs. combined compounds.

Table 2 presents descriptive statistics for OC nonusers (70.3%) and OC users (29.7%). Compared to OC nonusers, OC users were younger, had a lower waist circumference, higher triglyceride concentrations, and lower HbA1c; they reported fewer depressive symptoms (BDI–II) and less childhood trauma (CTQ); and on average, their blood was drawn 17 min earlier (Table 2). Importantly, OC users had drastically higher cortisol concentrations (Table 2).

**Figure 1:**
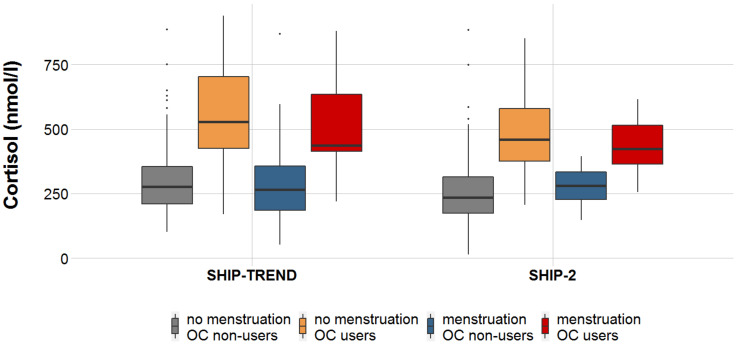
Boxplots are presented with the bold line symbolizing median serum cortisol concentrations and boxes summarizing the interquartile range. Whiskers represent concentrations lower than 25% and higher than 75%, respectively. Dots represent statistical outliers (M ± 3SD). Serum cortisol concentrations dependent on menstrual bleeding at the time of assessment and OC use are presented in both cohorts. Compared with nonmenstruating OC nonusers, both nonmenstruating and menstruating OC users had statistically higher cortisol concentrations in SHIP–TREND (nonmenstruating OC users: β = 0.551, t = 9.721, *p* = 1.02 × 10^−18^; menstruating OC users: β = 0.160, t = 2.638, *p* = 0.009) and SHIP–2 (nonmenstruating OC users: β = 0.475, t = 6.576, *p* = 8.32 × 10^−10^; menstruating OC users: β = 0.186, t = 4.939, *p* = 2.15 × 10^−6^). However, no statistical differences were observed between nonmenstruating and menstruating OC nonusers in SHIP–TREND (β = 0.022, t = 0.433, *p* = 0.665) or SHIP–2 (β = 0.035, t = 0.688, *p* = 0.492).

In women taking OCs, almost twofold higher blood cortisol concentrations were observed (OC nonusers: mean (M) = 287.29 nmol/l; OC users: M = 514.81 nmol/l). The reference concentrations of the ADVIA Centaur System in serum samples taken from 249 healthy individuals from 7:00 a.m. to 09:00 a.m. ranged from 118.6nmol/l to 618.0 nmol/l, meaning that the cortisol concentrations of women taking OCs still lay within the reference range. Interestingly, cortisol concentrations of OC users with menstrual bleeding at the time of assessment tended to be lower than in OC users without current menstrual bleeding (β = −0.139, t = −1.82, *p* = 0.072). Integrating menstrual bleeding and OC use (Figure 1), analyses demonstrated higher serum cortisol concentrations for OC use independent of current menstrual bleeding.

### 2.1. Associations between OC Intake and Blood Metabolite Concentrations

**Figure 2:**
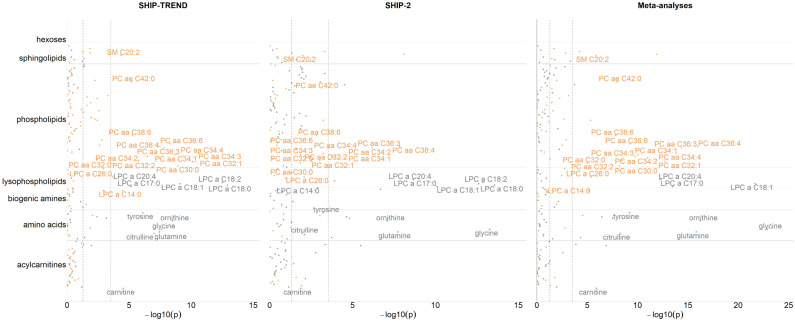
Main effects of OC use on the concentrations of the metabolites, including cohort–specific and meta–analytic results. Labeled metabolites were Bonferroni–corrected statistically significant in SHIP–TREND. The Bonferroni–corrected significance level (*p* < 2.924 × 10^−4^) is represented by the right dashed line, and the nominal significance level (*p* < 0.05) by the left dashed line in each panel. Analyses were adjusted for age, time of blood sampling, fasting time (restricted cubic splines), waist circumference, white blood cell count, red blood cell count, thrombocytes count and cystatin C concentrations. Gray = metabolite concentrations lower in OC users than in OC nonusers; yellow = metabolite concentrations higher in OC users than in OC nonusers.

Table 3 and Figure 2 summarize the results of the associations between OC use and metabolites. Technical coefficients of the analyzed metabolites are presented in Table S3. In SHIP–TREND, women taking OCs had higher plasma concentrations of 12 phosphatidylcholines with diacyl residue (PC aa C30:0, PC aa C32:0, PC aa C32:1, PC aa C32:2, PC aa C34:1, PC aa C34:2, PC aa C34:3, PC aa C34:4, PC aa C36:3, PC aa C36:4, PC aa C36:6, PC aa C38:6), higher concentrations of phosphatidylcholine with acyl–alkyl residue C42:0 (PC ae C42:0), lower concentrations of 5 lysophosphatidylcholines with acyl residue (LPC a C17:0, LPC a C18:0, LPC a C18:1, LPC a C18:2, LPC a C20:4), higher concentrations of LPC a C14:0 and LPC a C26:0, lower concentrations of 5 amino acids (citrulline, glutamine, glycine, ornithine, tyrosine), a lower concentration of carnitine and a higher concentration of the sphingomyelin (SM) C20:2. Besides PC aa C30:0 and LPC a C14:0, all results were replicated in SHIP–2. However, PC aa C30:0 reached Bonferroni–corrected significance in the meta–analytic results. Concentrations for the 27 metabolites associated with OC use in SHIP–TREND separated for both cohorts dependent on OC use and menstrual bleeding at the time of assessment are presented in Figure S1.

For the subsample of 114 SHIP–TREND women with available insulin concentrations, sensitivity analyses were calculated including insulin as a covariate. Only the association between OC use and PC aa C38:6 turned insignificant. Moreover, the effect of OC use on 15 (PC aa C30:0, PC aa C32:1, PC aa C32:2, PC aa C34:2, PC aa C34:3, PC aa C34:4, PC aa C36:3, LPC a C17:0, LPC a C18:0, LPC a C18:1, LPC a C18:2, citrulline, glutamine, glycine, ornithine) out of 27 metabolites remained significant even on a Bonferroni–corrected level.

### 2.2. Mediation Effects of Cortisol

**Figure 3:**
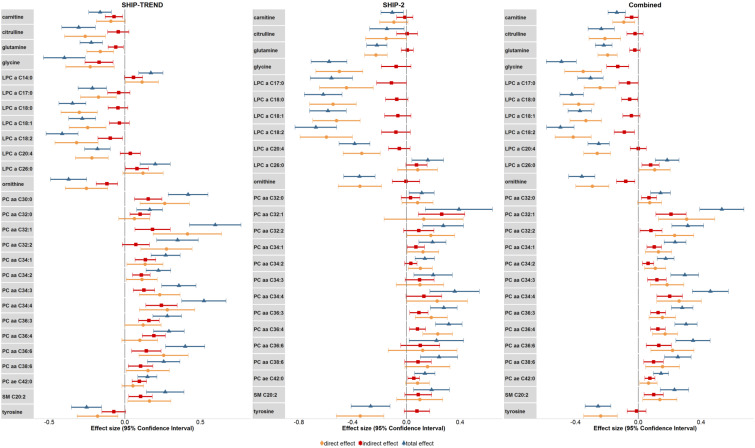
Statistical mediation of the OC effect on metabolites by cortisol. Analyses were adjusted for age, time of blood sampling, fasting time (restricted cubic splines), waist circumference, white blood cell count, red blood cell count, thrombocytes count and cystatin C concentrations (and cohort for the combined sample). Direct effect = OC effect on metabolites. Indirect effect = OC effect mediated through cortisol effect. Total effect = direct + indirect effect.

In the next step, we tested whether the above–reported effects in association to OC use were statistically mediated by cortisol. Associations between the metabolites and serum cortisol concentrations are presented in Table S4. In the meta–analysis for cohort–separated results, all metabolites associated with OC intake in SHIP–TREND (Table 3) were also associated with serum cortisol concentrations in the meta–analytic results (Table S4). Only the association between LPC a C14:0 concentrations and serum cortisol concentrations did not reach the Bonferroni–corrected significance level. The meta–analysis revealed that the effects of OC intake on carnitine (38.8% mediated), glycine (23.3% mediated) and ornithine (20.3% mediated) were significantly mediated by cortisol. Further, all 13 PCs (23.9%–60.4%) showed a significant indirect effect, as did LPC a C17:0 (19.6% mediated), LPC a 18:0 (12.1% mediated), LPC a 18:2 (15.8% mediated), LPC a C26:0 (40.2% mediated) and SM C20:2 (39.3% mediated). After a Bonferroni correction of the significance level, a significant indirect effect was observed for glycine, PCs except for PC aa C 32:0 and PC aa C32:2, and SM C20:2 (Figure 3). For an overview of the mediation effects and a breakdown of the cohort–specific results, see Table 4.

### 2.3. Extended Analyses To Assess the Impact of Menstrual Bleeding

To investigate the impact of menstrual bleeding on the associations between OC use and metabolite concentrations, the four–stage OC–menstruation variable (Figure 1) was used. OC nonusers without menstrual bleeding at the time of assessment were used as the reference group. The results of the cohort–separated analyses (Tables S5 and S6) and meta–analysis (Table S7) are presented in the supplementary material.

Except for LPC a C 14:0, the significant impact of OC use on metabolite concentrations was replicated for all metabolites, when comparing OC users and OC nonusers both without menstrual bleeding at the time of assessment via a meta–analysis of SHIP–TREND and SHIP–2. No significant differences were observed between OC nonusers without and with menstrual bleeding in the metabolome. Thus, OC use but not menstrual bleeding was associated with altered metabolite levels. Note, however, that the number of OC users with menstrual bleeding at the time of assessment was very low (SHIP–TREND: N = 10; SHIP–2: N = 7), limiting the statistical power to detect differences. For an overview of these results, see Table S7.

To investigate the impact of menstrual bleeding at the time of assessment on the mediation effect of cortisol, mediation analyses were run again while excluding women with menstrual bleeding at the time of assessment. Note, however, that the statistical power of these analyses is lower due to the reduced sample sizes (SHIP–TREND: N = 190, SHIP–2: N = 128). Results are presented in Table S8.

The meta–analysis based only on women without menstrual bleeding at the time of assessment replicated the significant indirect effects of OC intake reported for the whole sample (Table 4 and Table S8). After Bonferroni correction, a significant indirect effect was observed for almost all PCs (PC aa C 32:1, PC aa 34:1, PC aa C34:3, PC aa C34:4, PC aa C 36:3, PC aa C36:4 and PC ae C42:0) and LPC a C 18:2 (Table S8).

## 3. Discussion

The present study was based on two independent, large population–based cohorts to investigate associations between OC intake and blood metabolites and to investigate a potential mediation of these associations by cortisol. The main result demonstrated an association of OC intake with a targeted set of metabolites measured via Absolute*IDQ* p180 Kit among premenopausal women, including higher PC concentrations and lower LPC, amino acids, SM C20:2 and carnitine concentrations in the two independent cohorts. Note that both saturated and unsaturated PCs and LPCs were included. In a second step, we further provided evidence that cortisol concentrations, which are higher in OC users, might account for some of these associations including PCs, SM C20:2 and some amino acids, but not for the effects related to the LPCs. These observations support the hypothesis that an OC–use–induced increase in serum cortisol might contribute to the adverse metabolic side effects seen in OC users.

In search of an explanation for the association between OC intake and serum cortisol concentrations, we note that estrogens have been shown to upregulate omental and thigh 11β–hydroxysteroid dehydrogenase type 1 (11β–HSD1) mRNA expression in preadipocytes in women [31]. 11β–HSD1 is the enzyme that converts inactive cortisone to active cortisol. Thus, the observed increase of cortisol in OC users may be caused by estrogen–mediated changes in 11β–HSD1 activity. Interestingly, 11β–HSD1 has also been positively correlated with plasma triglycerides [32], indicating a possible linkage between OCs, elevated cortisol concentrations and plasma triglycerides. Moreover, cortisol has been associated with fatty liver and visceral fat in women [33,34]. Fatty liver is characterized by high secretion of triglycerides. In this context, an adverse effect of OC intake reported often is weight gain [35,36,37]. Finally, OC use has been related to higher triglyceride concentrations [4,38], a finding replicated in this study.

Accordingly, the present study observed replicable and broad associations between OC intake and plasma concentrations of various metabolites, including higher concentrations of glycerophospholipids, as well as lower concentrations of amino acids in OC users compared to OC nonusers. We provide evidence that these associations were largely mediated by the OC–associated increase in cortisol concentrations. However, since some PCs are part of the monolayer surrounding low–density lipoprotein particles, the alterations in glycerophospholipids might also refer to a triglyceride signature. Moreover, different forms of high– and low–density lipoproteins have different impacts on the lipidome. Nevertheless, the present work focused on the associations between OC intake and metabolome, although it is of high importance to extend our results to consider alterations in the lipidome.

Glucocorticoids have been shown to inhibit the activities of the phospholipase A2 and phospholipase C enzymes responsible for PC degradation in rat ovaries and spleen, and thus could be a reason for the elevated concentrations of PCs observed in the OC users [39,40,41]. Inline, in the present study, elevated concentrations of PCs were almost completely mediated by cortisol. On the other hand, the class of LPCs (PC–degradation products) showed lower concentrations in OC users. Interestingly, the statistical effects of OCs on LPCs, which may be a result of lower phospholipase activity [42], were mostly not significantly mediated by cortisol, indicating that another mechanism might play a more important role. However, LPCs are a heterogeneous group of fatty–acid derivatives and are implicated in a wide range of physiological and pathophysiological processes such as atherosclerosis, inflammation and activation of endothelial cells. In general, lower concentrations of LPCs are considered to be protective against thrombotic events [43,44,45], but whether LPCs are pro– or antiatherogenic depends on the fatty acid chain length and degree of saturation [46], complicating the interpretation of LPC signatures in the blood.

The measuring platform used in the present study, the Biocrates Absolute*IDQ* p180 kit, can only establish the total number of C–atoms and double bonds of the connected fatty acid. Thus, the presented data is insufficient to give fine–graded insights into the association patterns of different LPCs, PCs or SMs. In conclusion, this study delivered robust evidence that lipid signatures in the blood are altered in dependency on OCs. These changes deserve further attention to clarify whether they may play a role in the adverse effects of oral contraceptives.

Within the class of amino acids, we reported above that OC use was associated with lower concentrations of glutamine, glycine, tyrosine, citrulline, ornithine and carnitine. These effects were observed to be partly mediated by cortisol. It is noteworthy that ornithine and citrulline are intermediates of the urea cycle. In studies conducted on fetal rat livers or young rats, it was shown that glucocorticoids enhance the activity of certain enzymes of the urea cycle, which could lead to a higher turnover of those amino acids, and therefore potentially lower their plasma concentrations [47,48,49]. Additionally, in human studies, it has been proposed that progesterone itself has catabolic downstream effects and enhances amino–acid utilization throughout the whole female cycle of OC users, whereas a higher amino–acid turnover was only seen around the time of ovulation in OC nonusers [50,51]. Although the doses in the human experiments were higher than doses contained in OCs, the present results supported these findings of higher utilization of circulating amino acids.

Cortisol is a hormone secreted as part of the stress response of the body. As a consequence, energy substrates such as glucose, amino acids, glycerol and fatty acids are liberated during states of chronic stress to satisfy an elevated demand for energy substrates in the human body to maintain homeostasis [52]. Hence, metabolic changes in line with cortisol alterations are likely. The present study observed that altered metabolome concentrations due to OC intake were partly mediated by elevated cortisol levels, which might indicate an interaction of cortisol and the metabolome as part of the human stress response. OC intake was found to drastically enhance serum cortisol levels [9,10,11], replicated in the present study, and to mimic physical alterations induced by chronic stress [9]. However, a broader understanding of chronic stress mechanisms is needed to integrate the present findings. In this respect, in rodents, chronic unpredictable stress changed the composition of certain fatty–acid lipids and their biological properties in different brain regions [53], inducing alteration in redox states and elevating proinflammatory cytokines [54]. In general, chronic stress has been related to elevated cortisol concentrations in humans [55] and induced depression–like behavior in rodents [54]. Additionally, changes in serum cortisol concentrations and cortisol–binding–globulin (CBG) induced by altered estrogen concentrations in mice [56] and women [57,58] were linked to depression [59,60].

Consistent with the associations between cortisol concentrations and OC intake, the use of OC has been associated with a higher risk of depression and higher use of antidepressant medication, especially in adolescents [12,61,62]. A history of psychiatric symptoms strengthened the effects [35]. Moreover, sex hormones such as estradiol have been reported to influence brain development during puberty [63].

Hence, OC use does not only affect the stress axis, but also metabolic processes in the brain and periphery, which might impact the hormonal and neurological development during adolescents, as well as physical and mental health in adulthood. Interestingly, OC use and diseases connected with hypercortisolism share common side effects like type 2 diabetes mellitus, lower bone mineral density, hypertension, potential weight gain, and cardiovascular and mood disorders [12,64,65,66,67,68,69,70,71,72]. This interconnection highlights the importance to understand the mode of action of OCs to identify women with enhanced risk of suffering from these conditions.

Finally, the present results gave hint to the assumption that the effects of OC intake might still last for some time even after stopping the intake. Thus, effects in OC users with menstrual bleeding and hence pausing the intake at the time of assessment were hard to distinguish from OC users without menstrual bleeding at the time of assessment. However, the effect sizes to test these differences were very small. Larger studies and studies including women who shortly quit OC use are needed to validate these preliminary results.

Although the present study demonstrated metabolomic alterations associated with OC intake in two independent cohorts, it is important to note some limitations. The study cannot establish causality due to the observational design, missing interventional or longitudinal data. On a statistical level, the differences in fasting time between the two cohorts were not optimal, as fasting led to biochemical changes in a nonlinear manner. However, fasting time was modeled nonlinearly via restricted cubic splines, and cohort–separated results were integrated with meta–analyses to overcome this limitation. Nevertheless, it is unclear how differences in dynamic patterns such as fasting–related biochemical changes translate into statistical patterns in one–time metabolome measurement [73]. To enable mechanistic insights, a pharmaco–metabolomic study design would be more suitable, comparing the metabolome before OC use and at different time points after the medication intake. According to the manufacturer, the cross–reactivity of cortisol with cortisone and 11–deoxycortisol was 31% and 23%, respectively. Both cortisone and 11–deoxycortisol are biochemically related to cortisol. Hence, the cortisol concentrations may be overestimated. Nevertheless, an earlier study based on mass spectrometry to determine cortisol concentrations observed similar associations between OC use and cortisol concentrations [9]. Another limitation is the possibility of a selection bias, meaning that women who experience heavy side effects under OC treatment may interrupt their intake, and therefore they were not included as OC users in our data. The effect of long–term OC intake versus short–term OC intake also could not be evaluated. Hence, whether the changes in metabolite concentrations take place from the beginning or are established gradually over several cycles cannot be inferred from the presented data sets. Additionally, the sample size of women taking OCs was too small to evaluate any potential influences of different drug compositions on the blood metabolome. In particular, due to the small number of women taking progesterone–only pills, the present study could only evaluate the effect of combined oral contraceptives. Other hormone concentrations of potential relevance, e.g., prolactin, testosterone and estrogen, were not available in the present study. In sensitivity analyses, we adjusted the models for the SHIP–TREND cohort for insulin, but the results remained largely the same. However, future research is needed to validate our results in light of alterations in other hormone systems related to OC intake. Finally, only oral application of contraceptives was analyzed. Hence, results should not be generalized to implants and transdermal applications due to possible differences induced by the variegating pharmacokinetic attributes of transdermals and implants.

In conclusion, the present study is the first step to a comprehensive metabolic characterization of OC intake. We demonstrated systemic effects of OC intake on the metabolome in two general population cohorts. Within an approach to individualized medicine, the metabolic signature of OC use and an individual metabolic profile in OC users could help to identify women at higher risk of experiencing adverse effects and thus to prevent OC–induced stresses and implement alternative contraceptive methods as soon as possible. However, further investigation is needed to gain a more detailed understanding of the metabolic consequences of OC intake and thus to guarantee a safe long–term intake.

## 4. Materials and Methods

### 4.1. Study Population

Data of the Study of Health in Pomerania (SHIP) was used, comprising individuals from northeastern Germany [74]. The first baseline cohort (SHIP–0, N = 4308) was drawn from local registries and examined from 1997 until 2001. In parallel to the 11–year follow–up (SHIP–2, 2008–2012, N = 2333), a second, independent baseline cohort was drawn from virtually the same region and examined (SHIP–TREND, 2008–2012, N = 4420). Note that there was no participant overlap between these two cohorts. In both cohorts, only a subset of participants had metabolome characterizations (SHIP–2: *n* = 1547; SHIP–TREND: *n* = 991). For the present analyses, premenopausal, nonpregnant women with metabolome characterization and full covariate information aged 20–55 years were included from SHIP–TREND (*n* = 233) and SHIP–2 (*n* = 160). Two women were excluded due to transdermal and subcutaneous utilization of contraceptives (SHIP–2: *n* = 1; SHIP–TREND: *n* = 1).

The institutional review board of the University of Greifswald approved the survey and methods of the SHIP studies, and all analyses followed the Declaration of Helsinki. Written informed consent was provided by all participants.

### 4.2. Interview and Psychometric Data

A computer–assisted face–to–face interview was used to assess the medical history and sociodemographic factors of the study participants. Afterward, every participant underwent an extensive medical examination, including measurements of waist circumference, body height and weight. Since the body mass index (BMI) is biased by muscle mass [75], waist circumference was used as an indicator of abdominal fat in all analyses. Participants were asked to bring their medication prescription sheets or packing containers of all medication they had taken within the last seven days. All medications were recorded according to the Anatomical Therapeutic Chemical (ATC) classification. OCs were classified as ATC codes starting with G03AA, G03AB and G03AC [76]. Self–reported questionnaires were used to assess current menstrual bleeding or pregnancy. Women reporting pregnancy or absence of menstrual bleeding were excluded from the present analyses, as were women older than 65 years of age.

In SHIP–2, the Beck Depression Inventory–II (BDI–II), which is a 21–item self–reported questionnaire, was used to determine current depressive symptoms. It has high reliability as well as validity [77]. In SHIP–TREND, the PHQ–9 was used, which is a 9–item self–reported questionnaire assessing the diagnostic criteria of depression according to the DSM–IV. To ensure comparability of the BDI–II and PHQ–9, the PHQ–9 was transformed into the BDI–II according to Wahl et al. (2014). Briefly, PHQ–9 and BDI–II were made comparable via an item response theory (IRT)–based approach. Based on given PHQ–9 responses, responses to the BDI–II items were estimated and converted back into PHQ–9 with a correlation of r = 0.98 [78]. Finally, in both SHIP–TREND and SHIP–2, exposure to traumatic experiences was assessed with the Childhood Trauma Questionnaire (CTQ), which consists of 28 items rated on a 5–point Likert scale. Higher scores indicate higher exposure to traumatic events [79].

### 4.3. Blood Measurements

Blood samples were drawn between 7:00 a.m. and 12:30 p.m. Fasting time was assessed by asking for the last time the participants ate or drank beverages other than water. In SHIP–TREND, participants were explicitly asked not to eat or drink before blood sampling. Hence, fasting time was much longer in SHIP–TREND (M = 13:28 h, standard deviation (SD) = 1:32 h) than in SHIP–2 (M = 4:49 h, SD = 4:40 h). Blood samples were taken from the cubital vein and analyzed directly or stored at −80 °C in the Integrated Research Biobank of the University Medicine Greifswald [80,81]. White blood cell count (WBC), red blood cell count (RBC) and thrombocytes count (PLT) were measured on the XE 5000 from Sysmex (Sysmex Deutschland GmbH, Norderstedt, Germany). Glycated hemoglobin (HbA1c) concentrations were determined by high–performance liquid chromatography (Bio–Rad Diamat, Munich, Germany). Triglycerides were determined enzymatically (Dimension VISTA, Siemens Healthcare Diagnostics GmbH, Eschborn, Germany). For the processing of the serum samples, primary receptacles (BD Vacutainer Serum) with clot activator were used. The samples were centrifuged for 10 min at 1300× *g* at 18 °C. Afterward, aliquots were generated and frozen at −80 °C. To measure the cortisol concentrations, the aliquots were thawed for 20 min at room temperature, then vortexed for 10 s, and finally centrifuged for 3 min at 16,000× *g*. Serum cortisol concentrations were determined using a competitive immunoassay technique (ADVIA Centaur XP System, Siemens Healthcare Diagnostics, Eschborn, Germany). The manufacturer provided the cross–reactivity for 33 endogenous steroids, including corticosterone and 20–α– and 20–β–dihydrocortisol. Only two cross–reactivity values were reported to be larger than 10%: cortisone (31.1%) and 11–deoxycortisol (100 μg/dl; 23.3%). The assays were performed by skilled personnel according to the manufacturer’s instructions. For a subsample of 1000 SHIP–TREND participants, multiomic assessments were conducted, including insulin measurements. Of these participants, 114 premenopausal women were included in the present analyzed sample (37 OC users). Insulin concentrations were assessed using an electrochemiluminescence immunoassay (ADVIA Centaur, Siemens Healthcare Diagnostics, Eschborn, Germany).

### 4.4. Metabolomic Profiling

Using the Absolute*IDQ* p180 Kit (Biocrates Life Sciences AG, Innsbruck, Austria), targeted metabolomic profiling of the serum samples was performed. First, 10 µl aliquots of each plasma sample were processed in a fully automated assay as recommended by the manufacturer. Combining flow injection analysis (FIA) and liquid chromatography–mass spectrometry/mass spectrometry (LC–MS/MS), selective detection using multiple reaction monitoring–ion pairs, up to 188 metabolites from 5 different compound classes could be quantified. Via FIA, acylcarnitines, phospholipids and sphingolipids were measured in positive ionization mode; the sum of hexoses was measured in negative ionization mode. Using the LC–MS/MS method with an Agilent C18 column, amino acids and biogenic amines were detected. MS analyses were performed on an AB SCIEX 5500 QTrap™ mass spectrometer (AB SCIEX, Darmstadt, Germany) with electrospray ionization combined with an HPLC system (Agilent 1260 Infinity Binary LC, Santa Clara, CA, USA) including a degasser unit, column oven, autosampler and a binary pump. Internal standards (isotope–labeled) were partially integrated into the Kit plate for metabolite quantification. Afterward, data were preprocessed with Analyst software (Version 1.5.1, AB SCIEX, Darmstadt, Germany) including peak integration and concentration determination from calibration curves. Preprocessed data were uploaded into the Biocrates MetIDQ software, and the metabolite concentrations were automatically calculated.

As SHIP–2 and SHIP–TREND were independent cohorts and the metabolome measurements were performed separately in each cohort, differences in data–processing strategies occurred.

In SHIP–TREND, since samples were selected from the entire population at random, we used a methodology based on plate medians instead to account for plate differences. Thus, for each plate, the measured concentrations of the metabolites were divided by the median concentration, leading to equal median values for each metabolite on each plate. Subsequently, the median of the plate medians was calculated to reset to the original scale (µM concentrations). No obvious pattern in missing values became obvious. Only metabolites that were successfully measured in at least 20% of the entire population were taken forward, resulting in 183 metabolites.

In SHIP–2, an inhouse pool of plasma samples was utilized for assessing measurement quality. Briefly, external quality material, i.e., pooled plasma samples from two healthy male volunteers, was measured together with the study samples on each plate and used as the reference. To account for plate variations, the measured concentrations of the metabolites were divided by the median concentration of these quality controls for each metabolite on each plate. Afterward, the median of the plate medians from the quality controls was used to reset to the original scale. Coefficients of variations are reported in Table S3. Only metabolites that were successfully measured in at least 20% of the entire population were taken forward, resulting in 177 metabolites.

After applying these normalization steps, measurements were scaled to have a mean of 0 and a standard variation of 1 to make those comparable across both studies.

A principal component analysis was performed to detect multivariate outliers in both cohorts. These were defined as samples deviating more than 3 times the standard deviation from the mean Mahalanobis distance based on the first 10 principal components. As a result, 4 samples of the SHIP–TREND cohort and 22 samples of the SHIP–2 cohort were excluded. Finally, metabolite levels were log2–transformed.

Finally, only metabolites with more than 80% valid values in the study cohorts were used in the current statistical analyses (Table S3). The overlap of metabolites included in both cohorts was 171.

### 4.5. Statistical Analyses

Cohort–separated analyses were run via STATA 14.2; fixed–effect meta–analyses were run via R 3.6.2 using the metafor package [82]. Metric variables were described by M and SD, while categorical variables were described by proportions. First, we compared OC users and OC nonusers regarding a core set of variables used in similar analyses previously [9] (age, waist circumference, triglycerides, time of blood sampling, blood cell counts, glycated hemoglobin, BDI–II, CTQ and cortisol) to explore potential differences. To this end, we used Welch t–tests for metric variables and Fisher’s exact test for categorical variables.

Additionally, we investigated the association of cortisol concentrations with OC use and menstrual bleeding at the time of assessment via linear regression analyses. In this regard, we fitted linear regression models with the cortisol concentrations as the response variable. The predictors of interest used were: OC use (binary: yes/no), menstrual bleeding (binary: yes/no) and a four–staged OC–menstruation–variable categorizing (1) OC nonusers without menstruation, (2) OC users without menstruation, (3) OC nonusers with menstruation and (4) OC users with menstruation. Age, waist circumference, time of blood sampling, WBC, RBC, PLT, cystatin C concentrations (as an indicator for kidney function) and fasting time were included as covariates. As biochemical changes due to fasting are not linear over fasting time, restricted cubic splines (RCS) [83] with four knots (5th, 35th, 65th and 95th percentiles) were used to modulate these nonlinear relations in metabolites due to fasting time. Table S3 includes the *p*–values of fasting time on all metabolite levels. All analyses were calculated separately for both cohorts.

#### 4.5.1. Associations between OC Intake and Blood Metabolite Concentrations

To explore our main hypothesis, the associations of OCs with blood metabolite concentrations measured by the Absolute*IDQ* p180 Kit, we fitted linear regressions utilizing the log2–transformed metabolite concentration as the response variable and OC use (binary: yes/no) as the predictor of interest, while adjusting for age, waist circumference, time of blood sampling, WBC, RBC, PLT, cystatin C concentrations and fasting time. Again, fasting time was modeled using RCS to allow for nonlinear associations. Women with missing metabolite levels were excluded from the specific regression model, leading to variegating sample sizes for each metabolite. No missing value imputation was performed. We utilized the SHIP–TREND cohort as the discovery cohort, adjusting the significance level for multiple testing by Bonferroni correction (171 tests, p_adj_ < 2.924 × 10^−4^). Subsequently, we attempted to replicate the analyses for the Bonferroni–corrected significant metabolites in the SHIP–2 cohort using the same covariate vector and model specifications as in SHIP–TREND. A *p*–value < 0.05 was considered to be significant in the validation cohort given a Bonferroni–corrected significant result for this metabolite in SHIP–TREND. Furthermore, we ran a fixed–effect meta–analysis via R to integrate the cohort–separated analyses and to investigate potential heterogeneity between the results. Significance in the meta–analyses was evaluated after Bonferroni correction. In all regressions, standard errors robust to heteroscedasticity were used.

#### 4.5.2. Mediation Effects of Cortisol

Finally, we ran mediation analyses, testing whether the effects of OCs on the blood metabolome were statistically mediated by cortisol concentrations as a second hypothesis. To this end, the metabolites were regressed on serum cortisol concentrations. Afterward, we utilized Sobel–Goodman mediation tests via the STATA function sgmediation [84] on all metabolites that were associated with OC use in SHIP–2 and SHIP–TREND after correction for multiple testing. In these analyses, the blood metabolite concentration was the response variable, cortisol concentrations were defined as the mediator variable and OC use was the predictor of interest. We adjusted for the same covariates as before. Confidence intervals (95% CI) for indirect effects and percentage mediated were calculated via bootstrapping with 2000 replications. The results of cohort–separated analyses were integrated by meta–analyses. As mediation analyses have lower power [85], significance was assessed based on the meta–analytic results after Bonferroni correction. Nevertheless, results of the mediation analyses for each cohort alone were reported as well, although we expect those analyses to be underpowered.

#### 4.5.3. Extended Analyses to Assess the Impact of Menstrual Bleeding

To estimate the influence of menstrual bleeding at the time of assessment, all analyses investigating the associations between OC use and blood metabolite concentrations were recalculated by using the four–staged OC–menstrual bleeding variable as the predictor of interest. Four women reporting the intake of monophasic OCs, and thus not pausing OC use after 21 days, were excluded from these analyses. Again, all analyses were run cohort–separated and integrated with meta–analyses. The set of covariates was unchanged. The mediation analyses were rerun additionally while excluding women reporting menstrual bleeding at the time of assessment irrespective of OC use.

## Figures and Tables

**Table 1:** Descriptive statistics for the SHIP–TREND and SHIP–2 study cohorts.

Variables	SHIP-TREND (N = 232)			SHIP-2 (N = 159)			
	miss. %	M	SD	miss. %	M	SD	*p*-Value
Age (years)	0.00	38.940	7.959	0.00	42.340	5.202	5.14 × 10^−7 a^
Cortisol (nmol/l)	0.43	378.477	191.299	0.43	319.569	156.745	9.72 × 10^−4 a^
Time of Blood Sampling (h:min)	0.00	9:17	0:55	0.00	9:31	0:58	0.017 ^a^
Fasting Time (h:min)	0.00	13:28	1:32	0.00	4:49	4:40	1.56 × 10^−54 a^
Waist circumference (cm)	0.00	79.835	11.588	0.00	79.209	10.344	0.576 ^a^
BMI	0.00	25.933	5.051	0.00	25.783	4.672	0.763 ^a^
Triglycerides (mmol/l)	0.00	1.173	0.601	0.00	1.237	0.741	0.368 ^a^
HbA1c (%)	0.00	4.917	0.480	0.00	4.987	0.517	0.176 ^a^
Glucose (mmol/l)	0.00	5.031	0.444	0.00	5.056	0.924	0.756 ^a^
RBC (Tpt/l)	0.00	4.435	0.312	0.00	4.427	0.301	0.803 ^a^
WBC (Gpt/l)	0.00	6.088	1.614	0.00	6.532	1.829	0.014 ^a^
PLT (Gpt/l)	0.00	246.427	52.371	0.00	253.270	56.298	0.225 ^a^
Cystatin C (mg/l)	0.00	0.629	0.073	0.00	0.614	0.094	0.094 ^a^
BDI–II	0.00	8.831	6.329	0.00	4.918	5.963	1.44 × 10^−9 a^
CTQ	13.79	32.658	10.457	0.00	32.246	10.501	0.712 ^a^
Menstruation (% Yes)	0.00	18.10		0.00	19.50		0.792 ^b^
OC use (% Yes)	0.00	31.47		0.00	27.04		0.369 ^b^

^a^*t*-Test, 2-sided *p*-value; ^b^ Fisher’s exact test, 2-sided *p*-value; miss. = missing values; M = mean; SD = standard deviation; BMI = body mass index; RBC = red blood cell count; WBC = white blood cell count; PLT = thrombocytes count; HbA1c = glycated hemoglobin; BDI-II = Beck Depression Inventory-II; CTQ = Childhood Trauma Questionnaire; Menstruation = menstrual bleeding at the time of assessment.

**Table 2:** Descriptive statistics of OC users and OC nonusers.

Variables	Nonusers (N = 275)			Users (N = 116)			
	miss. %	M	SD	miss. %	M	SD	*p*–Value
Age (years)	0.00	41.236	6.543	0.00	38.155	8.071	3.58 × 10^−4 a^
Cortisol (nmol/l)	0.36	287.289	136.503	0.86	514.808	170.651	1.04 × 10^−26 a^
Time of Blood Sampling (h:min)	0.00	9:28	0:58	0.00	9:11	0:52	0.003 ^a^
Fasting Time (h:min)	0.00	9:52	5:23	0.00	10:10	5:10	0.596 ^a^
Waist circumference (cm)	0.00	80.529	11.210	0.00	77.331	10.505	0.008 ^a^
BMI	0.00	26.145	4.978	0.00	25.226	4.649	0.082 ^a^
Triglycerides (mmol/l)	0.00	1.149	0.675	0.00	1.320	0.613	0.015 ^a^
HbA1c (%)	0.00	4.983	0.484	0.00	4.859	0.513	0.028 ^a^
Glucose (mmol/l)	0.00	5.038	0.536	0.00	5.049	0.941	0.907 ^a^
RBC (Tpt/l)	0.00	4.421	0.308	0.00	4.458	0.305	0.276 ^a^
WBC (Gpt/l)	0.00	6.279	1.754	0.00	6.243	1.631	0.844 ^a^
PLT (Gpt/l)	0.00	249.611	53.928	0.00	248.259	54.514	0.822 ^a^
Cystatin C (mg/l)	0.00	0.628	0.085	0.00	0.612	0.075	0.065 ^a^
BDI–II	0.00	7.954	7.021	0.00	5.547	4.508	6.58 × 10^−5 a^
CTQ	9.09	33.163	11.186	6.03	30.900	8.418	0.036 ^a^
Menstruation (% Yes)	0.00	20.36		0.00	14.66		0.203 ^b^

^a^*t*-Test, 2-sided *p*–value; ^b^ Fisher’s exact test, 2-sided *p*-value; OC = oral contraceptives; miss. = missing values; M = mean; SD = standard deviation; BMI = body mass index; RBC = red blood cell count; WBC = white blood cell count; PLT = thrombocytes count; HbA1c = glycated hemoglobin; BDI-II = Beck Depression Inventory–II; CTQ = Childhood Trauma Questionnaire; Menstruation = menstrual bleeding at the time of assessment.

**Table 3:** Metabolites associated with OC intake.

Metabolites	SHIP−TREND			SHIP−2			Meta−Analysis			Heterogeneity	
	b	95%−CI	*p*−Value	b	95%−CI	*p*−Value	b	95%−CI	*p*−Value	b	*p*−Value
Carnitine	−0.161	(−0.236; −0.087)	2.995 × 10^−5^	−0.107	(−0.191; −0.023)	0.013	−0.137	(−0.193; −0.082)	1.225 × 10^−6^	0.000	0.338
Citrulline	−0.304	(−0.413; −0.195)	9.932 × 10^−8^	−0.160	(−0.283; −0.036)	0.012	−0.241	(−0.322; −0.160)	5.719 × 10^−9^	66.637	0.083
Glutamine	−0.221	(−0.297; −0.145)	3.647 × 10^−8^	−0.221	(−0.295; −0.148)	1.833 × 10^−8^	−0.221	(−0.274; −0.169)	1.506 × 10^−16^	0.000	0.997
Glycine	−0.406	(−0.544; −0.268)	2.393 × 10^−8^	−0.567	(−0.702; −0.433)	5.219 × 10^−14^	−0.489	(−0.584; −0.393)	1.302 × 10^−23^	63.400	0.098
Ornithine	−0.374	(−0.493; −0.254)	3.262 × 10^−9^	−0.337	(−0.455; −0.219)	8.020 × 10^−8^	−0.355	(−0.438; −0.272)	5.973 × 10^−17^	0.000	0.666
Tyrosine	−0.252	(−0.350; −0.154)	8.019 × 10^−7^	−0.256	(−0.400; −0.112)	6.050 × 10^−4^	−0.253	(−0.334; −0.173)	6.590 × 10^−10^	0.000	0.968
*LPC a C14:0*	*0.172*	*(0.092; 0.252)*	*3.409 × 10^−5^*	*−0.022*	*(−0.074; 0.031)*	*0.418*	*0.036*	*(−0.007; 0.080)*	*0.101*	*93.705*	*6.733 × 10^−5^*
LPC a C17:0	−0.219	(−0.313; −0.125)	6.939 × 10^−6^	−0.547	(−0.701; −0.393)	7.979 × 10^−11^	−0.308	(−0.388; −0.229)	3.282 × 10^−14^	92.229	3.341 × 10^−4^
LPC a C18:0	−0.351	(−0.438; −0.264)	1.201 × 10^−13^	−0.602	(−0.743; −0.462)	2.657 × 10^−14^	−0.421	(−0.495; −0.348)	3.767 × 10^−29^	88.879	0.003
LPC a C18:1	−0.284	(−0.371; −0.197)	7.642 × 10^−10^	−0.566	(−0.704; −0.427)	2.438 × 10^−13^	−0.364	(−0.437; −0.290)	2.086 × 10^−22^	91.345	6.761 × 10^−4^
LPC a C18:2	−0.418	(−0.524; −0.312)	3.081 × 10^−13^	−0.650	(−0.807; −0.492)	1.371 × 10^−13^	−0.490	(−0.578; −0.403)	3.688 × 10^−28^	82.877	0.016
LPC a C20:4	−0.184	(−0.269; −0.099)	3.052 × 10^−5^	−0.378	(−0.494; −0.261)	2.136 × 10^−9^	−0.251	(−0.319; −0.183)	5.892 × 10^−13^	85.715	0.008
LPC a C26:0	0.198	(0.095; 0.302)	2.052 × 10^−4^	0.157	(0.041; 0.273)	0.008	0.180	(0.103; 0.257)	4.243 × 10^−6^	0.000	0.597
*PC aa C30:0*	*0.417*	*(0.288; 0.546)*	*1.151 × 10^−9^*	*0.113*	*(−0.040; 0.267)*	*0.147*	*0.291*	*(0.193; 0.389)*	*6.495 ×10^−9^*	*88.780*	*0.003*
PC aa C32:0	0.162	(0.076; 0.247)	2.387 × 10^−4^	0.113	(0.023; 0.202)	0.014	0.138	(0.077; 0.200)	9.959 × 10^−6^	0.000	0.432
PC aa C32:1	0.597	(0.425; 0.769)	7.411 × 10^−11^	0.376	(0.131; 0.621)	0.003	0.524	(0.384; 0.664)	1.977 × 10^−13^	53.103	0.144
PC aa C32:2	0.349	(0.210; 0.489)	1.649 × 10^−6^	0.255	(0.105; 0.404)	9.568 × 10^−4^	0.305	(0.204; 0.406)	3.649 × 10^−9^	0.000	0.361
PC aa C34:1	0.268	(0.175; 0.360)	3.861 × 10^−8^	0.193	(0.095; 0.292)	1.629 × 10^−4^	0.233	(0.166; 0.300)	1.020 × 10^−11^	15.374	0.277
PC aa C34:2	0.219	(0.137; 0.301)	3.159 × 10^−7^	0.138	(0.067; 0.208)	1.719 × 10^−4^	0.172	(0.119; 0.226)	1.947 × 10^−10^	54.672	0.137
PC aa C34:3	0.356	(0.244; 0.468)	2.084 × 10^−9^	0.185	(0.039; 0.330)	0.013	0.292	(0.204; 0.380)	9.071 × 10^−11^	70.558	0.065
PC aa C34:4	0.523	(0.377; 0.670)	2.720 × 10^−11^	0.333	(0.145; 0.521)	6.211 × 10^−4^	0.451	(0.336; 0.566)	1.510 × 10^−14^	59.707	0.115
PC aa C36:3	0.280	(0.186; 0.374)	1.562 × 10^−8^	0.269	(0.167; 0.371)	6.559 × 10^−7^	0.275	(0.206; 0.344)	4.254 × 10^−15^	0.000	0.877
PC aa C36:4	0.291	(0.191; 0.391)	3.364 × 10^−8^	0.305	(0.205; 0.405)	1.438 × 10^−8^	0.298	(0.227; 0.368)	1.061 × 10^−16^	0.000	0.841
PC aa C36:6	0.400	(0.270; 0.529)	4.941 × 10^−9^	0.202	(4.075 × 10^−4^; 0.403)	0.050	0.342	(0.233; 0.450)	5.853×10^−10^	62.569	0.102
PC aa C38:6	0.254	(0.147; 0.361)	5.319 × 10^−6^	0.229	(0.089; 0.369)	0.002	0.245	(0.160; 0.329)	1.464 × 10^−8^	0.000	0.779
PC ae C42:0	0.149	(0.085; 0.213)	6.620 × 10^−6^	0.129	(0.054; 0.205)	9.087 × 10^−4^	0.141	(0.093; 0.189)	1.107 × 10^−8^	0.000	0.694
SM C20:2	0.265	(0.140; 0.390)	4.368 × 10^−5^	0.174	(0.042; 0.306)	0.010	0.222	(0.132; 0.312)	1.412 × 10^−6^	0.000	0.326

Analyses adjusted for age, time of blood sampling, fasting time (restricted cubic splines), waist circumference, white blood cell count, red blood cell count, thrombocytes count and cystatin C concentrations. Metabolites with *p* < 2.924 × 10^−4^ (Bonferroni correction for 171 tests) in SHIP–TREND are presented. Italics = nominal significance in SHIP-2 (*p* < 0.05) not reached.

**Table 4:** Mediating effects of cortisol.

Metabolites	SHIP–TREND			SHIP–2			Meta–Analysis							Heterogeneity		
	ind. Effect	95%–CI	*p*–Value	ind. Effect	95%–CI	*p*–Value	ind. Effect	95%–CI	*p*–Value	% Mediated	95%–CI	*p*–Value	ind. Effect	*p*–Value	% Mediated	*p*–Value
*Carnitine*	*−0.071*	*(−0.129; −0.013)*	*0.016*	*−0.012*	*(−0.072; 0.049)*	*0.708*	*−0.043*	*(−0.085; −8.483 × 10^−4^)*	*0.046*	*38.8*	*(−132.074; 209.589)*	*0.657*	*48.391*	*0.164*	*0.0*	*0.894*
*Citrulline*	*−0.043*	*(−0.113; 0.027)*	*0.232*	*0.006*	*(−0.072; 0.084)*	*0.890*	*−0.021*	*(−0.073; 0.031)*	*0.427*	*14.1*	*(−10.725; 38.872)*	*0.266*	*0.000*	*0.366*	*0.0*	*0.973*
*Glutamine*	*−0.059*	*(−0.110; −0.009)*	*0.022*	*0.007*	*(−0.038; 0.053)*	*0.759*	*−0.022*	*(−0.056; 0.011)*	*0.195*	*9.2*	*(−7.389; 25.752)*	*0.277*	*72.704*	*0.056*	*67.3*	*0.080*
Glycine	−0.170	(−0.263; −0.077)	3.329 × 10^−4^	−0.077	(−0.187; 0.034)	0.173	−0.131	(−0.202; −0.060)	2.919 × 10^−4^	23.3	(6.980; 39.549)	0.005	37.887	0.204	64.0	0.096
*Ornithine*	*−0.118*	*(−0.190; −0.047)*	*0.001*	*−0.003*	*(−0.106; 0.099)*	*0.953*	*−0.081*	*(−0.139; −0.022)*	*0.007*	*20.3*	*(1.739; 38.868)*	*0.032*	*69.378*	*0.071*	*59.8*	*0.115*
*Tyrosine*	*−0.073*	*(−0.151; 0.006)*	*0.070*	*0.078*	*(−0.017; 0.173)*	*0.106*	*−0.011*	*(−0.072; 0.049)*	*0.717*	*9.3*	*(−20.772; 39.385)*	*0.544*	*82.659*	*0.016*	*68.6*	*0.075*
*LPC a C17:0*	*−0.040*	*(−0.114; 0.034)*	*0.293*	*−0.111*	*(−0.222; −0.001)*	*0.048*	*−0.062*	*(−0.124; −5.429 × 10^−4^)*	*0.048*	*19.6*	*(1.022; 38.258)*	*0.039*	*10.691*	*0.290*	*0.0*	*0.949*
*LPC a C18:0*	*−0.045*	*(−0.111; 0.021)*	*0.179*	*−0.072*	*(−0.156; 0.011)*	*0.090*	*−0.055*	*(−0.107; −0.004)*	*0.035*	*12.1*	*(0.474; 23.752)*	*0.041*	*0.000*	*0.614*	*0.0*	*0.918*
*LPC a C18:1*	*−0.036*	*(−0.102; 0.031)*	*0.293*	*−0.064*	*(−0.162; 0.035)*	*0.204*	*−0.044*	*(−0.099; 0.011)*	*0.114*	*11.5*	*(−2.964; 25.864)*	*0.119*	*0.000*	*0.643*	*0.0*	*0.913*
*LPC a C18:2*	*−0.096*	*(−0.178; −0.014)*	*0.021*	*−0.078*	*(−0.185; 0.029)*	*0.154*	*−0.089*	*(−0.154; −0.024)*	*0.007*	*15.8*	*(2.619; 28.971)*	*0.019*	*0.000*	*0.794*	*0.0*	*0.408*
*LPC a C20:4*	*0.037*	*(−0.031; 0.105)*	*0.288*	*−0.053*	*(−0.134; 0.027)*	*0.196*	*−5.618 × 10^−4^*	*(−0.052; 0.051)*	*0.983*	*7.0*	*(−12.660; 26.673)*	*0.485*	*64.309*	*0.094*	*45.4*	*0.176*
*LPC a C26:0*	*0.081*	*(0.005; 0.157)*	*0.037*	*0.075*	*(−0.004; 0.154)*	*0.062*	*0.078*	*(0.023; 0.133)*	*0.005*	*40.2*	*(−13.432; 93.807)*	*0.142*	*0.000*	*0.919*	*0.0*	*0.988*
*PC aa C32:0*	*0.101*	*(0.034; 0.168)*	*0.003*	*0.031*	*(−0.039; 0.101)*	*0.388*	*0.068*	*(0.019; 0.116)*	*0.006*	*60.4*	*(−13.577; 134.281)*	*0.110*	*50.930*	*0.153*	*0.0*	*0.867*
PC aa C32:1	0.184	(0.067; 0.300)	0.002	0.262	(0.090; 0.435)	0.003	0.208	(0.112; 0.305)	2.387 × 10^−5^	31.2	(7.617; 54.729)	0.009	0.000	0.460	0.0	0.733
*PC aa C32:2*	*0.073*	*(−0.017; 0.163)*	*0.110*	*0.092*	*(−0.021; 0.205)*	*0.110*	*0.080*	*(0.010; 0.151)*	*0.025*	*23.9*	*(−2.330; 50.108)*	*0.074*	*0.000*	*0.797*	*0.0*	*0.689*
PC aa C34:1	0.137	(0.068; 0.205)	9.963 × 10^−5^	0.071	(0.006; 0.135)	0.031	0.101	(0.054; 0.148)	2.335 × 10^−5^	49.6	(17.485; 81.697)	0.002	47.150	0.169	0.0	0.831
PC aa C34:2	0.109	(0.051; 0.168)	2.305 × 10^−4^	0.033	(−0.013; 0.079)	0.158	0.062	(0.026; 0.098)	7.017 × 10^−4^	40.0	(11.929; 68.131)	0.005	75.473	0.043	0.0	0.399
PC aa C34:3	0.127	(0.056; 0.198)	4.660 × 10^−4^	0.098	(−0.010; 0.206)	0.075	0.118	(0.059; 0.178)	9.501 × 10^−5^	35.5	(11.077; 59.937)	0.004	0.000	0.663	0.0	0.979
PC aa C34:4	0.243	(0.139; 0.347)	4.848 × 10^−6^	0.130	(−0.003; 0.263)	0.055	0.200	(0.118; 0.282)	1.721 × 10^−6^	44.3	(22.211; 66.454)	8.571 × 10^−5^	41.377	0.192	0.0	0.720
PC aa C36:3	0.160	(0.092; 0.228)	4.283 × 10^−6^	0.093	(0.026; 0.160)	0.007	0.126	(0.078; 0.174)	2.511 × 10^−7^	43.3	(21.085; 65.441)	1.317 × 10^−4^	46.161	0.173	3.2	0.309
PC aa C36:4	0.193	(0.116; 0.269)	7.582 × 10^−7^	0.082	(0.022; 0.143)	0.007	0.125	(0.077; 0.172)	2.334 × 10^−7^	36.9	(19.170; 54.588)	4.472 × 10^−5^	79.710	0.026	73.7	0.051
PC aa C36:6	0.143	(0.046; 0.239)	0.004	0.103	(−0.042; 0.248)	0.164	0.130	(0.050; 0.211)	0.002	35.7	(7.449; 63.917)	0.013	0.000	0.657	0.0	0.984
PC aa C38:6	0.104	(0.023; 0.186)	0.012	0.086	(−0.013; 0.185)	0.088	0.097	(0.034; 0.160)	0.002	38.5	(−0.517; 77.553)	0.053	0.000	0.779	0.0	0.900
PC ae C42:0	0.097	(0.049; 0.144)	7.618 × 10^−5^	0.055	(0.011; 0.098)	0.015	0.074	(0.041; 0.106)	7.957 × 10^−6^	55.4	(21.783; 89.112)	0.001	38.023	0.204	0.0	0.488
SM C20:2	0.104	(0.026; 0.182)	0.009	0.088	(−0.012; 0.188)	0.084	0.098	(0.037; 0.160)	0.002	39.3	(−2.271; 80.930)	0.064	0.000	0.800	0.0	0.944

Adjusted for age, time of blood sampling, fasting time (restricted cubic splines), waist circumference, white blood cell count, red blood cell count, thrombocytes count and cystatin C concentrations. Metabolites presented if the effect of OC use was Bonferroni–corrected significant (171 tests: *p* < 2.924 × 10^−4^) in SHIP–TREND. LPC a C 14:0 and PC aa 30:0 are missing due to nonsignificant associations between these metabolite concentrations and cortisol in SHIP-2 (Table S4). Italics = indirect effect in meta–analysis after Bonferroni correction for mediation analyses (25 tests. *p* < 0.002) were not significant.

## Data Availability

The data presented in this study are not publicly available due to the data use policy of SHIP. All SHIP data can be applied for free of charge from the SHIP transfer office (https://www.fvcm.med.uni–greifswald.de/cm_antrag/index.php).

## Supplementary Materials

The following are available online at https://www.mdpi.com/2218-1989/11/4/193/s1, Table S1: Distribution of the oral contraceptive compounds in SHIP–TREND, Table S2: Distribution of the oral contraceptive compounds in SHIP–2, Table S3: Technical descriptive statistics for the analyzed metabolites in SHIP–TREND and SHIP–2, Table S4: Metabolites associated with serum cortisol concentrations, Table S5: Metabolites associated with OC intake in the dependence of menstrual bleeding at the time of assessment in SHIP–TREND, Table S6: Metabolites associated with OC intake in the dependence of menstrual bleeding at the time of assessment in SHIP–2, Table S7: Meta–analytic results for metabolites associated with OC intake in the dependence of menstrual bleeding at the time of assessment, Table S8: Mediating effects of cortisol in women without menstrual bleeding at the time of assessment. Figure S1: Boxplots are presented with the bold line symbolizing median serum cortisol concentrations and boxes summarizing the interquartile range. Whiskers are presenting concentrations lower than 25% and higher than 75%, respectively. Dots are representing statistical outliers (M ± 3SD). Metabolite concentrations dependent on menstrual bleeding at the time of assessment and OC use are presented in both cohorts. Metabolites are presented if the effect of OC use was Bonferroni–corrected significant (171 tests: *p* < 2.924 × 10^−4^) in SHIP–TREND.
