# Supplementary material for: Broad Metabolome Alterations Associated with the Intake of Oral Contraceptives Are Mediated by Cortisol in Premenopausal Women
Source: Metabolites. 2021 Mar 24;11(4):193. doi: 10.3390/metabo11040193 (PMC8064380; doi:10.3390/metabo11040193)
Supplement: Supplementary file 1 [file metabolites-11-00193-s001.pdf]

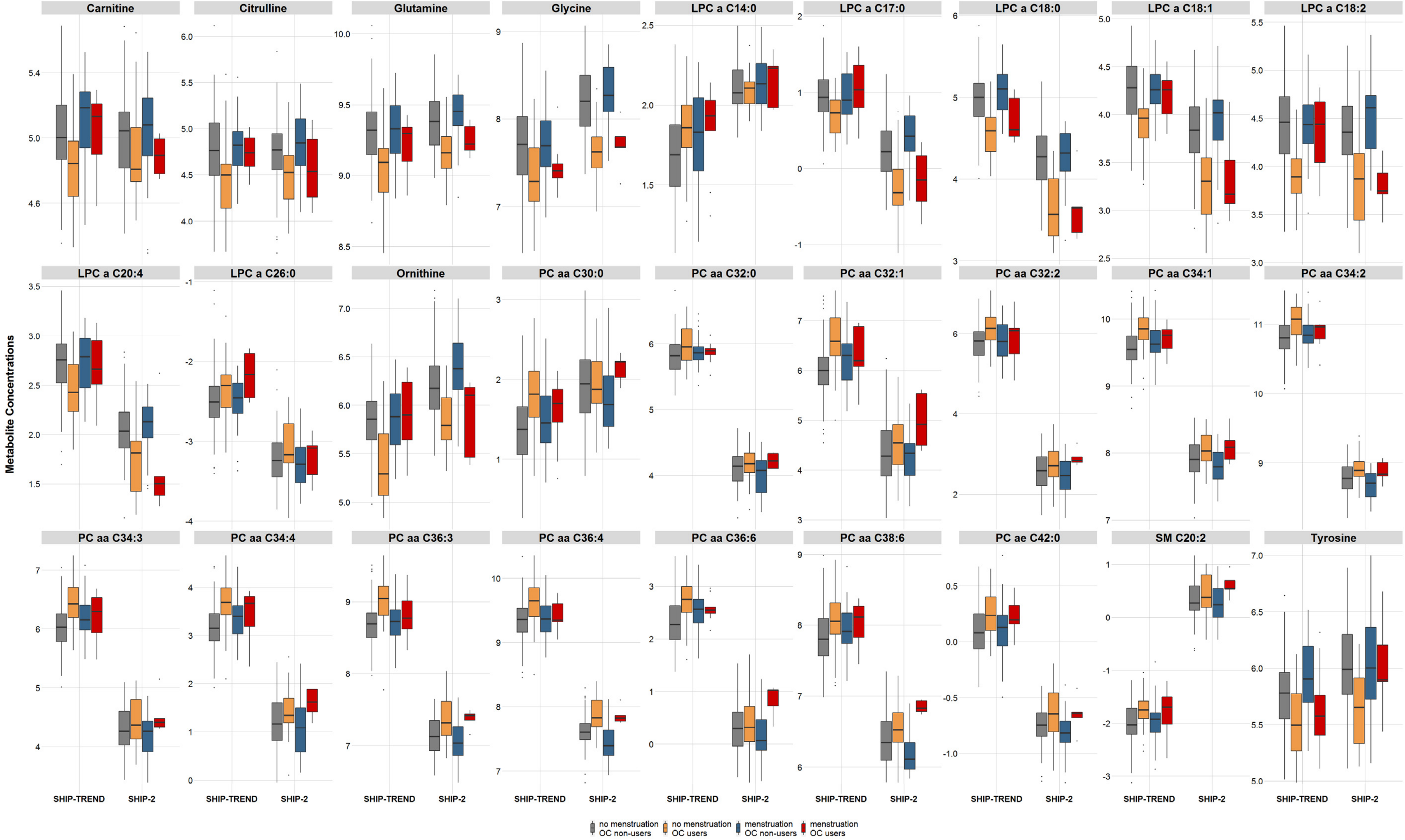

Carnitine
Citrulline
Glutamine
Glycine
LPC a C14:0
LPC a C17:0
LPC a C18:0
LPC a C18:1
LPC a C18:2
LPC a C20:4
LPC a C26:0
Ornithine
PC aa C30:0
PC aa C32:0
PC aa C32:1
PC aa C32:2
PC aa C34:1
PC aa C34:2
PC aa C34:3
PC aa C34:4
PC aa C36:3
PC aa C36:4
PC aa C36:6
PC aa C38:6
PC ae C42:0
SM C20:2
Tyrosine
Metabolite Concentrations
SHIP-TREND
SHIP-2
no menstruation OC non-users
no menstruation OC users
menstruation OC non-users
menstruation OC users

Figure S1. Boxplots are presented with the bold line symbolizing median serum cortisol concentrations and boxes summarizing the interquartile range. Whiskers are presenting concentrations lower than 25% and higher than 75%, respectively. Dots are representing statistical outliers (M±3SD). Metabolite concentrations dependent on menstrual bleeding at the time of assessment and OC use are presented in both cohorts. Metabolites are presented if the effect of OC use was Bonferroni-corrected significant (171 tests: $p < 2.924 \times 10^{-4}$) in SHIP-TREND.

Table S1: Distribution of the oral contraceptive compounds in SHIP-TREND.

Active Ingredient		Dosage (mg)		N	%
		Ethinylestradiol	Progesterone		
**SINGLE COMPOUND**					
	**Desogestrel**		0.075	3	4.1
**COMBINED COMPOUND**					
Monophasic					
**Ethinylestradiol**	**Norgestimate**	0.035	0.25	3	4.1
	**Drospirenone**	0.02	3	4	5.5
		0.03	3	3	4.1
	**Dienogest**	0.03	2	15	20.5
	**Levonorgestrel**	0.02	0.1	4	5.5
		0.03	0.125	8	11.0
		0.03	0.15	4	5.5
		0.05	0.125	2	2.7
	**Desogestrel**	0.02	0.15	10	13.7
		0.03	0.15	2	2.7
	**Chlormadinonaceta**	0.03	2	3	4.1
Triphasic					
**Ethinylestradiol**	**Levonorgestrel**	0.03	0.125	11	15.1
	**Desogestrel**	0.035	0.05	1	1.4

*as specified in column three

Table S2: Distribution of the oral contraceptive compounds in SHIP-2.

Active Ingredient		Dosage (mg)		N	%
		Ethinylestradio	Progesterone		
**SINGLE COMPOUND**					
	**Desogestrel**		0.075	1	2.3
**COMBINED COMPOUND**					
Monophasic					
**Ethinylestradiol**	**Drospirenone**	0.02	3	1	5
		0.03	3	3	7.0
	**Dienogest**	0.03	2	5	11.6
	**Levonorgestrel**	0.02	0.1	1	2.3
		0.03	0.125	8	18.6
		0.03	0.15	6	14.0
	**Desogestrel**	0.02	0.15	6	14.0
		0.03	0.15	2	4.7
	**Chlormadinonaceta**	0.03	2	1	2.3
Step-up-pill					
**Ethinylestradiol**	**Chlormadininacetat**	0.05	1	2	4.7
Triphasic					
**Ethinylestradiol**	**Levonorgestrel**	0.03	0.05	5	11.6
	**Norethisteron**	0.035	1	1	2.3

**Norgestimat**	0.035	0.18	1	2.3

*as specified in column three

Table S3: Technical descriptive statistics for the analyzed metabolites in SHIP-TREND and SHIP-2.

		**SHIP-TREND**		**SHIP-2**		
		**% above LOD**	**p-value fasting**	**CV**	**% above LOD**	**p-value fasting**
**Acylcarnitines**	**Carnitine**	99.14	0.950	6.55	100.00	0.950
	**Propionylcarnitine**	98.28	0.995	5.92	100.00	0.995
	**Hydroxybutyrylcarnitine**	98.71	0.147	15.58	98.11	0.147
	**Hydroxypropionylcarnitine**	98.71	0.476	15.13	98.74	0.476
	**Propenoylcarnitine**	99.57	0.691	15.29	98.74	0.691
	**Butyrylcarnitine**	99.14	0.268	8.01	99.37	0.268
	**Butenylcarnitine**	99.14	0.748	16.70	99.37	0.748
	**Glutarylcarnitine**	99.14	0.296	21.14	99.37	0.296
	**Methylglutarylcarnitine**	99.14	0.898	11.23	99.37	0.898
	**Hydroxyvalerylcarnitine**	97.84	0.936	9.38	98.74	0.936
	**Tiglylcarnitine**	99.57	0.898	8.96	96.86	0.898
	**Glutaconylcarnitine**	99.14	0.650	13.67	99.37	0.650
	**Pimelylcarnitine**	100.00	0.252	11.04	100.00	0.252
	**Octanoylcarnitine**	98.71	0.743	10.56	100.00	0.743
	**Nonaylcarnitine**	99.14	0.945	9.66	99.37	0.945
	**Decanoylcarnitine**	99.57	0.311	14.77	100.00	0.311
	**Decenoylcarnitine**	99.57	0.499	8.62	98.74	0.499
	**Decadienylcarnitine**	99.14	0.667	8.88	100.00	0.667
	**Dodecanoylcarnitine**	98.71	0.256	9.19	100.00	0.256
	**Dodecanedioylcarnitine**	99.14	0.809	7.74	99.37	0.809
	**Dodecenoylcarnitine**	99.57	0.060	6.59	100.00	0.060
	**Tetradecanoylcarnitine**	98.28	0.170	22.48	99.37	0.170
	**Tetradecenoylcarnitine**	99.57	0.047	15.53	99.37	0.047
	**Hydroxytetradecenoylcarnitine**	99.57	0.861	18.33	98.74	0.861
	**Tetradecadienylcarnitine**	100.00	0.081	15.21	99.37	0.081
	**Hydroxytetradecadienylcarnitine**	99.57	0.747	24.66	100.00	0.747
	**Hexadecanoylcarnitine**	98.71	0.155	9.02	99.37	0.155
	**Hydroxyhexadecanoylcarnitine**	98.71	0.174	19.69	100.00	0.174
	**Hexadecenoylcarnitine**	100.00	0.059	14.71	100.00	0.059
	**Hydroxyhexadecenoylcarnitine**	99.57	0.946	22.46	97.48	0.946
	**Hexadecadienylcarnitine**	99.14	0.504	23.66	98.74	0.504
	**Octadecanoylcarnitine**	98.71	0.278	16.95	100.00	0.278
	**Octadecenoylcarnitine**	99.14	0.004	9.43	100.00	0.004
	**Hydroxyoctadecenoylcarnitine**	99.14	0.402	18.59	100.00	0.402
	**Octadecadienylcarnitine**	100.00	0.047	18.16	98.74	0.047

% above LOD = % above limit of detection, fasting = p-value of non-linear associations with fasting time (restricted cubic splines); CV = coefficient of variance

Note: CVs were not available for SHIP-TREND as no technical samples were measured for replication.

Table S1 - continued: Technical descriptive statistics for the analyzed metabolites in SHIP-TREND and SHIP-2

		SHIP-TREND		SHIP-2		
		% above LOD	p-value fasting	CV	% above LOD	p-value fasting
Amino Acids	Alanine	99.14	0.648	5.13	100.00	0.648
	Arginine	97.84	0.336	6.65	100.00	0.336
	Asparagine	97.41	0.829	4.48	100.00	0.829
	Aspartate	98.28	0.312	11.92	94.97	0.312
	Citrulline	97.84	0.040	4.14	100.00	0.040
	Glutamine	98.28	0.945	7.99	98.74	0.945
	Glutamate	99.14	0.257	6.55	98.74	0.257
	Glycine	99.14	0.656	5.91	99.37	0.656
	Histidine	98.28	0.385	6.08	98.11	0.385
	Isoleucine	99.14	0.079	5.22	100.00	0.079
	Leucine	99.14	0.444	5.49	100.00	0.444
	Lysine	97.84	0.153	7.79	100.00	0.153
	Methionine	99.14	0.050	7.46	100.00	0.050
	Ornithine	97.41	0.916	8.64	97.48	0.916
	Phenylalanine	98.71	0.625	5.75	100.00	0.625
	Proline	99.14	0.724	5.89	98.11	0.724
	Serine	98.28	0.932	7.16	99.37	0.932
	Threonine	98.28	0.027	4.45	100.00	0.027
	Tryptophan	99.14	0.004	5.38	99.37	0.004
	Tyrosine	98.28	0.914	5.27	98.11	0.914
	Valine	99.57	0.280	5.48	99.37	0.280
Biogenic Amines	Acetylornithine	98.28	0.212	8.80	96.86	0.212
	Asymmetric dimethylarginine	86.64	0.684	8.79	99.37	0.684
	Alpha-aminoadipic acid	91.81	0.442	14.95	96.23	0.442
	Creatinine	99.57	0.390	3.00	99.37	0.390
	Kynurenine	98.71	0.416	8.03	100.00	0.416
	Methioninesulfoxide	95.69	0.475	8.21	97.48	0.475
	Serotonin	87.93	0.968	9.10	95.60	0.968
	Spermidine	94.40	0.969	3.64	99.37	0.969
	Spermine	86.21	0.100	7.56	97.48	0.100
	Hydroxyproline	99.14	0.139	6.08	91.19	0.139
	Taurine	98.71	0.423	3.32	99.37	0.423
	Total dimethylarginine	94.40	0.633	5.07	99.37	0.633

% above LOD = % above limit of detection, fasting = p-value of non-linear associations with fasting time (restricted cubic splines); CV = coefficient of variance

Note: CVs were not available for SHIP-TREND as no technical samples were measured for replication.

Table S1 - continued: Technical descriptive statistics for the analyzed metabolites in SHIP-TREND and SHIP-2

		SHIP-TREND		SHIP-2		
		% above LOD	p-value fasting	CV	% above LOD	p-value fasting
Lysophospholipids	**LPC a C14:0**	99.57	0.269	6.72	99.37	0.269
	**LPC a C16:0**	99.57	0.345	6.86	100.00	0.345
	**LPC a C16:1**	100.00	0.944	6.23	99.37	0.944
	**LPC a C17:0**	99.57	0.833	7.08	99.37	0.833
	**LPC a C18:0**	98.28	0.243	7.26	97.48	0.243
	**LPC a C18:1**	99.57	0.864	6.88	100.00	0.864
	**LPC a C18:2**	99.57	0.552	6.76	100.00	0.552
	**LPC a C20:3**	98.71	0.824	6.23	100.00	0.824
	**LPC a C20:4**	99.57	0.202	5.76	100.00	0.202
	**LPC a C24:0**	98.71	0.033	12.52	100.00	0.033
	**LPC a C26:0**	99.14	0.053	14.20	100.00	0.053
	**LPC a C26:1**	100.00	0.077	12.02	100.00	0.077
	**LPC a C28:0**	99.14	0.025	12.71	100.00	0.025
	**LPC a C28:1**	100.00	0.573	8.34	100.00	0.573
Phospholipids	**PC aa C24:0**	98.28	0.367	11.96	98.11	0.367
	**PC aa C26:0**	98.71	0.032	5.79	100.00	0.032
	**PC aa C28:1**	100.00	0.473	6.07	100.00	0.473
	**PC aa C30:0**	99.57	0.111	5.53	100.00	0.111
	**PC aa C32:0**	99.14	0.383	4.44	100.00	0.383
	**PC aa C32:1**	98.71	0.498	4.75	100.00	0.498
	**PC aa C32:2**	99.57	0.072	4.99	99.37	0.072
	**PC aa C32:3**	99.14	0.923	6.27	100.00	0.923
	**PC aa C34:1**	99.14	0.398	5.66	100.00	0.398
	**PC aa C34:2**	98.71	0.498	7.52	100.00	0.498
	**PC aa C34:3**	99.14	0.509	5.32	98.74	0.509
	**PC aa C34:4**	99.14	0.674	5.06	99.37	0.674
	**PC aa C36:0**	100.00	0.552	11.31	100.00	0.552
	**PC aa C36:1**	99.14	0.555	4.89	100.00	0.555
	**PC aa C36:2**	99.14	0.658	6.33	100.00	0.658
	**PC aa C36:3**	97.84	0.795	5.12	100.00	0.795
	**PC aa C36:4**	98.71	0.910	5.69	99.37	0.910
	**PC aa C36:5**	99.57	0.167	5.63	99.37	0.167
	**PC aa C36:6**	99.57	0.943	6.09	99.37	0.943
	**PC aa C38:0**	100.00	0.266	5.10	100.00	0.266

% above LOD = % above limit of detection, fasting = p-value of non-linear associations with fasting time (restricted cubic splines); CV = coefficient of variance

Note: CVs were not available for SHIP-TREND as no technical samples were measured for replication.

Table S1 - continued: Technical descriptive statistics for the analyzed metabolites in SHIP-TREND and SHIP-2

		SHIP-TREND		SHIP-2		
		% above LOD	p-value fasting	CV	% above LOD	p-value fasting
Phospholipids	PC aa C38:1	92.67	0.744	22.56	98.74	0.744
	PC aa C38:3	100.00	0.468	4.96	100.00	0.468
	PC aa C38:4	99.57	0.470	5.23	99.37	0.470
	PC aa C38:5	100.00	0.856	5.62	99.37	0.856
	PC aa C38:6	100.00	0.341	4.69	100.00	0.341
	PC aa C40:1	99.57	0.476	6.74	100.00	0.476
	PC aa C40:2	99.14	0.699	9.09	100.00	0.699
	PC aa C40:3	99.14	0.316	8.13	100.00	0.316
	PC aa C40:4	99.14	0.431	5.19	100.00	0.431
	PC aa C40:5	100.00	0.809	5.70	100.00	0.809
	PC aa C40:6	100.00	0.527	5.01	100.00	0.527
	PC aa C42:0	99.14	0.004	7.28	99.37	0.004
	PC aa C42:1	99.14	0.560	6.20	100.00	0.560
	PC aa C42:2	99.57	0.515	7.39	99.37	0.515
	PC aa C42:4	99.14	0.257	7.85	100.00	0.257
	PC aa C42:5	99.57	0.472	7.92	100.00	0.472
	PC aa C42:6	99.14	0.079	6.91	100.00	0.079
	PC ae C30:0	99.57	0.028	5.25	100.00	0.028
	PC ae C30:2	100.00	0.431	6.15	100.00	0.431
	PC ae C32:1	99.57	0.227	5.36	100.00	0.227
	PC ae C32:2	99.14	0.255	8.97	100.00	0.255
	PC ae C34:0	99.57	0.019	5.53	100.00	0.019
	PC ae C34:1	99.57	0.117	4.98	100.00	0.117
	PC ae C34:2	99.57	0.091	5.17	100.00	0.091
	PC ae C34:3	99.57	0.721	4.60	100.00	0.721
	PC ae C36:0	99.57	0.764	6.35	99.37	0.764
	PC ae C36:1	99.57	0.049	5.25	100.00	0.049
	PC ae C36:2	99.14	0.070	5.57	100.00	0.070
	PC ae C36:3	99.57	0.480	5.26	100.00	0.480
	PC ae C36:4	99.57	0.339	4.47	100.00	0.339
	PC ae C36:5	100.00	0.686	5.53	100.00	0.686
	PC ae C38:0	99.57	0.671	5.69	100.00	0.671
	PC ae C38:2	99.14	0.717	7.33	100.00	0.717
	PC ae C38:3	99.57	0.495	4.67	99.37	0.495
	PC ae C38:4	99.57	0.334	5.29	100.00	0.334
	PC ae C38:5	99.14	0.740	4.72	100.00	0.740

% above LOD = % above limit of detection, fasting = p-value of non-linear associations with fasting time (restricted cubic splines); CV = coefficient of variance

Note: CVs were not available for SHIP-TREND as no technical samples were measured for replication.

Table S1 - continued: Technical descriptive statistics for the analyzed metabolites in SHIP-TREND and SHIP-2

		SHIP-TREND		SHIP-2		
		% above LOD	p-value fasting	CV	% above LOD	p-value fasting
Phospholipids	**PC ae C38:6**	100.00	0.522	4.91	100.00	0.522
	**PC ae C40:1**	99.57	0.978	6.07	100.00	0.978
	**PC ae C40:2**	99.57	0.823	5.41	100.00	0.823
	**PC ae C40:3**	98.71	0.579	5.82	100.00	0.579
	**PC ae C40:4**	99.57	0.386	4.51	99.37	0.386
	**PC ae C40:5**	100.00	0.728	4.43	100.00	0.728
	**PC ae C40:6**	100.00	0.386	5.41	100.00	0.386
	**PC ae C42:0**	98.71	0.599	7.33	100.00	0.599
	**PC ae 42:1**	100.00	0.439	6.60	100.00	0.439
	**PC ae C42:2**	99.57	0.903	7.65	100.00	0.903
	**PC ae C42:3**	100.00	0.927	6.14	100.00	0.927
	**PC ae C42:4**	99.57	0.034	4.68	98.74	0.034
	**PC ae C42:5**	99.57	0.140	3.93	98.11	0.140
	**PC ae C44:3**	99.14	0.152	12.65	100.00	0.152
	**PC ae C44:4**	99.14	0.636	7.86	98.11	0.636
	**PC ae C44:5**	100.00	0.108	6.30	98.11	0.108
	**PC ae C44:6**	99.57	0.247	4.96	98.74	0.247
Sphingolipids	**SM (OH) C14:1**	100.00	0.365	4.74	99.37	0.365
	**SM (OH) C16:1**	100.00	0.371	5.51	100.00	0.371
	**SM (OH) C22:1**	100.00	0.249	5.38	100.00	0.249
	**SM (OH) C22:2**	100.00	0.273	6.22	100.00	0.273
	**SM (OH) C24:1**	98.71	0.282	6.14	100.00	0.282
	**SM C16:0**	100.00	0.560	5.41	100.00	0.560
	**SM C16:1**	100.00	0.073	5.31	100.00	0.073
	**SM C18:0**	99.57	0.029	5.27	100.00	0.029
	**SM C18:1**	99.14	0.259	4.94	100.00	0.259
	**SM C20:2**	99.14	0.158	7.29	98.74	0.158
	**SM C22:3**	91.81	0.229	9.62	100.00	0.229
	**SM C24:0**	100.00	0.045	5.35	100.00	0.045
	**SM C24:1**	100.00	0.016	5.17	100.00	0.016
	**SM C26:0**	98.71	0.280	8.15	100.00	0.280
	**SM C26:1**	99.57	0.403	12.84	100.00	0.403
	**Hexose**	98.28	0.590	5.16	98.74	0.590

% above LOD = % above limit of detection, fasting = p-value of non-linear associations with fasting time (restricted cubic splines); CV = coefficient of variance

Note: CVs were not available for SHIP-TREND as no technical samples were measured for replication.

Table S4: Metabolites associated with serum cortisol concentrations.

	SHIP-TREND			SHIP-2			Meta-analysis			Heterogeneity	
	**b**	**95%-CI**	**p-value**	**b**	**95%-CI**	**p-value**	**b**	**95%-CI**	**p-value**	**b**	**p-value**
**Carnitine**	-4.761x10^{-4}	(-6.692x10^{-4};-2.831x10^{-4})	2.236x10^{-6}	-2.341x10^{-4}	(-5.032x10^{-4};3.507x10^{-5})	0.088	-3.936x10^{-4}	(-5.494x10^{-4};-2.378x10^{-4})	7.413x10^{-7}	51.971	0.149
**Citrulline**	-5.975x10^{-4}	(-8.798x10^{-4};-3.153x10^{-4})	4.378x10^{-5}	-2.334x10^{-4}	(-6.041x10^{-4};1.374x10^{-4})	0.216	-4.635x10^{-4}	(-6.866x10^{-4};-2.404x10^{-4})	4.671x10^{-5}	57.999	0.123
**Glutamine**	-5.259x10^{-4}	(-7.259x10^{-4};-3.260x10^{-4})	4.990x10^{-7}	-3.786x10^{-4}	(-6.261x10^{-4};-1.311x10^{-4})	0.003	-4.675x10^{-4}	(-6.220x10^{-4};-3.130x10^{-4})	3.004x10^{-9}	0.000	0.360
**Glycine**	-0.001	(-0.001;-8.022x10^{-4})	1.783x10^{-10}	-0.001	(-0.002;-8.178x10^{-4})	8.876x10^{-7}	-0.001	(-0.001;-9.164x10^{-4})	3.826x10^{-17}	0.000	0.534
**Ornithine**	-9.485x10^{-4}	(-0.001;-6.740x10^{-4})	9.655x10^{-11}	-6.759x10^{-4}	(-0.001;-2.411x10^{-4})	0.003	-8.705x10^{-4}	(-0.001;-6.400x10^{-4})	1.364x10^{-13}	8.765	0.295
**Tyrosine**	-6.198x10^{-4}	(-8.931x10^{-4};-3.465x10^{-4})	1.266x10^{-5}	-1.277x10^{-4}	(-5.430x10^{-4};2.876x10^{-4})	0.544	-4.705x10^{-4}	(-6.973x10^{-4};-2.437x10^{-4})	4.792x10^{-5}	73.829	0.051
LPC a C14:0	*4.437x10^{-4}*	*(2.356x10^{-4};6.517x10^{-4})*	*3.834x10^{-5}*	*-2.685x10^{-5}*	*(-2.071x10^{-4};1.534x10^{-4})*	*0.769*	*1.743x10^{-4}*	*(3.906x10^{-5};3.096x10^{-4})*	*0.012*	*91.210*	*7.439x10^{-4}*
**LPC a C17:0**	-4.465x10^{-4}	(-7.178x10^{-4};-1.752x10^{-4})	0.001	-0.001	(-0.002;-9.647x10^{-4})	1.119x10^{-8}	-6.961x10^{-4}	(-9.292x10^{-4};-4.630x10^{-4})	4.818x10^{-9}	92.298	3.143x10^{-4}
**LPC a C18:0**	-6.643x10^{-4}	(-9.060x10^{-4};-4.227x10^{-4})	1.598x10^{-7}	-0.001	(-0.002;-9.201x10^{-4})	3.366x10^{-9}	-8.332x10^{-4}	(-0.001;-6.250x10^{-4})	4.265x10^{-15}	86.824	0.006
**LPC a C18:1**	-5.393x10^{-4}	(-7.689x10^{-4};-3.097x10^{-4})	6.320x10^{-6}	-0.001	(-0.002;-8.612x10^{-4})	2.010x10^{-8}	-7.071x10^{-4}	(-9.083x10^{-4};-5.058x10^{-4})	5.764x10^{-12}	89.230	0.002
**LPC a C18:2**	-9.347x10^{-4}	(-0.001;-6.543x10^{-4})	3.665x10^{-10}	-0.002	(-0.002;-0.001)	1.752x10^{-9}	-0.001	(-0.001;-8.504x10^{-4})	3.646x10^{-19}	77.161	0.036
**LPC a C20:4**	-1.547x10^{-4}	(-4.142x10^{-4};1.048x10^{-4})	0.241	-8.989x10^{-4}	(-0.001;-5.227x10^{-4})	5.433x10^{-6}	-3.955x10^{-4}	(-6.077x10^{-4};-1.832x10^{-4})	2.601x10^{-4}	90.327	0.001
**LPC a C26:0**	5.609x10^{-4}	(2.965x10^{-4};8.252x10^{-4})	4.196x10^{-5}	5.904x10^{-4}	(2.447x10^{-4};9.361x10^{-4})	9.441x10^{-4}	5.718x10^{-4}	(3.632x10^{-4};7.804x10^{-4})	7.763x10^{-8}	0.000	0.893
**PC aa C30:0**	0.001	(7.810x10^{-4};0.001)	2.339x10^{-10}	4.910x10^{-4}	(-8.930x10^{-5};0.001)	0.097	9.588x10^{-4}	(6.743x10^{-4};0.001)	3.937x10^{-11}	70.226	0.067
**PC aa C32:0**	5.616x10^{-4}	(3.266x10^{-4};7.966x10^{-4})	4.416x10^{-6}	3.270x10^{-4}	(-4.301x10^{-6};6.583x10^{-4})	0.053	4.828x10^{-4}	(2.924x10^{-4};6.732x10^{-4})	6.732x10^{-7}	23.118	0.254
**PC aa C32:1**	0.001	(0.001;0.002)	1.403x10^{-12}	0.002	(9.947x10^{-4};0.003)	1.368x10^{-5}	0.002	(0.001;0.002)	2.264x10^{-18}	0.000	0.501
**PC aa C32:2**	7.518x10^{-4}	(4.197x10^{-4};0.001)	1.308x10^{-5}	8.632x10^{-4}	(3.174x10^{-4};0.001)	0.002	7.821x10^{-4}	(5.001x10^{-4};0.001)	5.451x10^{-8}	0.000	0.731
**PC aa C34:1**	8.289x10^{-4}	(5.993x10^{-4};0.001)	1.606x10^{-11}	6.332x10^{-4}	(3.423x10^{-4};9.240x10^{-4})	3.078x10^{-5}	7.535x10^{-4}	(5.745x10^{-4};9.325x10^{-4})	1.594x10^{-16}	7.978	0.297
**PC aa C34:2**	6.743x10^{-4}	(4.772x10^{-4};8.715x10^{-4})	1.411x10^{-10}	3.776x10^{-4}	(1.644x10^{-4};5.908x10^{-4})	6.157x10^{-4}	5.371x10^{-4}	(3.934x10^{-4};6.809x10^{-4})	2.421x10^{-13}	75.420	0.044
**PC aa C34:3**	9.363x10^{-4}	(6.641x10^{-4};0.001)	1.113x10^{-10}	7.509x10^{-4}	(2.596x10^{-4};0.001)	0.003	8.925x10^{-4}	(6.560x10^{-4};0.001)	1.415x10^{-13}	0.000	0.514
**PC aa C34:4**	0.002	(0.001;0.002)	5.193x10^{-15}	0.001	(5.761x10^{-4};0.002)	1.518x10^{-4}	0.001	(0.001;0.002)	3.126x10^{-20}	8.793	0.295
**PC aa C36:3**	9.143x10^{-4}	(6.915x10^{-4};0.001)	4.435x10^{-14}	8.741x10^{-4}	(5.589x10^{-4};0.001)	1.819x10^{-7}	9.008x10^{-4}	(7.201x10^{-4};0.001)	1.536x10^{-22}	0.000	0.837
**PC aa C36:4**	0.001	(7.799x10^{-4};0.001)	3.582x10^{-13}	8.961x10^{-4}	(5.972x10^{-4};0.001)	2.169x10^{-8}	9.795x10^{-4}	(7.821x10^{-4};0.001)	2.319x10^{-22}	0.000	0.460

**PC aa C36:6**	0.001	(6.973x10^{-4};0.001)	1.056x10^{-8}	8.198x10^{-4}	(1.591x10^{-4};0.001)	0.015	9.946x10^{-4}	(6.904x10^{-4};0.001)	1.467x10^{-10}	0.000	0.555
**PC aa C38:6**	7.162x10^{-4}	(4.300x10^{-4};0.001)	1.613x10^{-6}	7.830x10^{-4}	(3.090x10^{-4};0.001)	0.001	7.341x10^{-4}	(4.906x10^{-4};9.776x10^{-4})	3.435x10^{-9}	0.000	0.812
**PC ae C42:0**	5.246x10^{-4}	(3.565x10^{-4};6.928x10^{-4})	3.710x10^{-9}	4.666x10^{-4}	(2.649x10^{-4};6.682x10^{-4})	1.021x10^{-5}	5.007x10^{-4}	(3.725x10^{-4};6.290x10^{-4})	1.998x10^{-14}	0.000	0.662
**SM C20:2**	7.251x10^{-4}	(4.178x10^{-4};0.001)	5.761x10^{-6}	6.859x10^{-4}	(2.595x10^{-4};0.001)	0.002	7.117x10^{-4}	(4.640x10^{-4};9.594x10^{-4})	1.789x10^{-8}	0.000	0.883

Analyses adjusted for age, time of blood sampling, fasting time, waist circumference, white blood cell count, red blood cell count, thrombocytes count, and cystatin C concentrations. Metabolites are presented if the effect of OC use was Bonferroni-corrected significant (171 tests: $p < 2.924x10^{-4}$) in SHIP-TREND. Italics = meta-analytic effect after Bonferroni-correction (27 test: $p < 0.002$) not significant

Table S5. Metabolites associated with OC intake in the dependence of menstrual bleeding at the time of assessment in SHIP-TREND.

	no menstruation			menstruation					
	OC users (N = 63)			OC non-users (N = 32)			OC users (N = 10)		
	b	95%-CI	p-value	b	95%-CI	p-value	b	95%-CI	p-value
**Carnitine**	-0.184	(-0.264;-0.104)	$9.415x10^{-6}$	0.063	(-0.038;0.165)	0.221	0.025	(-0.172;0.222)	0.802
**Citrulline**	-0.337	(-0.461;-0.213)	$2.129x10^{-7}$	0.063	(-0.070;0.195)	0.351	-0.035	(-0.211;0.141)	0.697
**Glutamine**	-0.245	(-0.332;-0.158)	$8.266x10^{-8}$	0.030	(-0.063;0.124)	0.526	-0.076	(-0.200;0.047)	0.226
**Glycine**	-0.443	(-0.599;-0.287)	$6.479x10^{-8}$	0.022	(-0.142;0.187)	0.789	-0.286	(-0.503;-0.069)	0.010
**Ornithine**	-0.481	(-0.599;-0.363)	$7.152x10^{-14}$	0.009	(-0.121;0.138)	0.897	0.135	(-0.118;0.387)	0.296
**Tyrosine**	-0.279	(-0.380;-0.177)	$1.747x10^{-7}$	0.151	(0.030;0.271)	0.015	-0.028	(-0.298;0.243)	0.841
**LPC a C14:0**	0.202	(0.115;0.288)	$7.006x10^{-6}$	0.107	(-0.009;0.223)	0.069	0.241	(0.053;0.430)	0.012
**LPC a C17:0**	-0.277	(-0.377;-0.176)	$1.510x10^{-7}$	0.001	(-0.135;0.137)	0.987	0.064	(-0.173;0.301)	0.595
**LPC a C18:0**	-0.371	(-0.468;-0.275)	$1.043x10^{-12}$	0.119	(0.004;0.234)	0.042	-0.167	(-0.354;0.019)	0.079
**LPC a C18:1**	-0.325	(-0.423;-0.228)	$3.715x10^{-10}$	0.062	(-0.052;0.177)	0.283	-0.018	(-0.188;0.151)	0.831
**LPC a C18:2**	-0.476	(-0.594;-0.359)	$8.135x10^{-14}$	0.032	(-0.098;0.162)	0.629	-0.098	(-0.324;0.128)	0.393
**LPC a C20:4**	-0.202	(-0.296;-0.107)	$3.738x10^{-5}$	0.016	(-0.108;0.140)	0.797	-0.036	(-0.248;0.177)	0.741
**LPC a C26:0**	0.182	(0.066;0.298)	0.002	0.017	(-0.103;0.137)	0.779	0.359	(0.186;0.531)	$5.807x10^{-5}$
**PC aa C30:0**	0.512	(0.376;0.649)	$3.292x10^{-12}$	0.207	(0.030;0.383)	0.022	0.334	(0.070;0.598)	0.013
**PC aa C32:0**	0.204	(0.109;0.299)	$3.558x10^{-5}$	0.091	(-0.014;0.195)	0.089	0.134	(0.006;0.261)	0.040
**PC aa C32:1**	0.725	(0.541;0.908)	$2.816x10^{-13}$	0.255	(0.039;0.472)	0.021	0.364	(0.020;0.707)	0.038
**PC aa C32:2**	0.410	(0.263;0.557)	$1.060x10^{-7}$	0.084	(-0.106;0.275)	0.385	0.255	(-0.136;0.645)	0.200
**PC aa C34:1**	0.317	(0.219;0.415)	$1.095x10^{-9}$	0.123	(0.004;0.242)	0.043	0.255	(0.022;0.488)	0.032
**PC aa C34:2**	0.271	(0.186;0.357)	$1.928x10^{-9}$	0.065	(-0.030;0.160)	0.180	0.152	(-0.048;0.351)	0.135
**PC aa C34:3**	0.455	(0.335;0.574)	$1.541x10^{-12}$	0.227	(0.075;0.379)	0.004	0.188	(-0.053;0.430)	0.126
**PC aa C34:4**	0.633	(0.485;0.781)	$5.059x10^{-15}$	0.204	(0.028;0.379)	0.023	0.439	(0.073;0.804)	0.019
**PC aa C36:3**	0.343	(0.246;0.440)	$4.116x10^{-11}$	0.052	(-0.065;0.170)	0.379	0.148	(-0.065;0.362)	0.171
**PC aa C36:4**	0.358	(0.252;0.464)	$2.183x10^{-10}$	0.039	(-0.089;0.168)	0.548	0.156	(-0.020;0.331)	0.081
**PC aa C36:6**	0.470	(0.331;0.609)	$2.276x10^{-10}$	0.213	(0.040;0.386)	0.016	0.419	(0.178;0.661)	$7.283x10^{-4}$
**PC aa C38:6**	0.278	(0.161;0.395)	$5.083x10^{-6}$	0.075	(-0.070;0.221)	0.308	0.297	(0.075;0.518)	0.009
**PC ae C42:0**	0.160	(0.087;0.233)	$2.396x10^{-5}$	-0.004	(-0.110;0.103)	0.948	0.111	(-0.004;0.227)	0.059
**SM C20:2**	0.292	(0.151;0.433)	$6.388x10^{-5}$	0.048	(-0.108;0.203)	0.547	0.322	(0.060;0.583)	0.016

Analyses adjusted for age, time of blood sampling, fasting time (RCS), waist circumference, white blood cell count, red blood cell count, thrombocytes count, and cystatin C concentrations. OC non-users without menstrual bleeding at the time of assessment used as the reference group (N = 127).
Metabolites presented if the effect of OC use was Bonferroni-corrected significant (171 tests: $p < 2.924x10^{-4}$) in SHIP-TREND.

Table S6. Metabolites associated with OC intake in the dependence of menstrual bleeding at the time of assessment in SHIP-2.

	no menstruation	menstruation	
	OC users	OC non-users	OC users

	(N = 36)			(N = 24)			(N = 7)		
	**b**	**95%-CI**	**p-value**	**b**	**95%-CI**	**p-value**	**b**	**95%-CI**	**p-value**
**Carnitine**	-0.114	(-0.211;-0.016)	0.023	0.007	(-0.139;0.154)	0.921	-0.061	(-0.173;0.052)	0.290
**Citrulline**	-0.163	(-0.295;-0.031)	0.016	0.100	(-0.061;0.261)	0.221	-0.002	(-0.324;0.320)	0.990
**Glutamine**	-0.225	(-0.309;-0.142)	$3.775x10^{-7}$	0.039	(-0.065;0.143)	0.459	-0.144	(-0.228;-0.060)	$9.311x10^{-4}$
**Glycine**	-0.545	(-0.694;-0.396)	$2.586x10^{-11}$	0.085	(-0.110;0.279)	0.392	-0.564	(-0.802;-0.327)	$6.104x10^{-6}$
**Ornithine**	-0.310	(-0.434;-0.185)	$2.291x10^{-6}$	0.144	(-0.043;0.332)	0.131	-0.265	(-0.643;0.114)	0.169
**Tyrosine**	-0.304	(-0.447;-0.161)	$4.595x10^{-5}$	0.073	(-0.141;0.287)	0.502	0.128	(-0.183;0.440)	0.418
**LPC a C14:0**	-0.019	(-0.076;0.038)	0.505	0.051	(-0.030;0.132)	0.213	0.034	(-0.081;0.150)	0.558
**LPC a C17:0**	-0.495	(-0.669;-0.320)	$1.025x10^{-7}$	0.208	(0.013;0.403)	0.037	-0.531	(-0.806;-0.255)	$2.062x10^{-4}$
**LPC a C18:0**	-0.559	(-0.716;-0.403)	$7.069x10^{-11}$	0.108	(-0.060;0.276)	0.205	-0.661	(-0.923;-0.400)	$1.691x10^{-6}$
**LPC a C18:1**	-0.507	(-0.658;-0.356)	$6.185x10^{-10}$	0.213	(0.018;0.407)	0.032	-0.578	(-0.860;-0.297)	$7.961x10^{-5}$
**LPC a C18:2**	-0.579	(-0.751;-0.406)	$5.957x10^{-10}$	0.131	(-0.062;0.324)	0.181	-0.843	(-1.179;-0.507)	$1.976x10^{-6}$
**LPC a C20:4**	-0.336	(-0.461;-0.211)	$4.120x10^{-7}$	0.100	(-0.056;0.256)	0.208	-0.458	(-0.722;-0.194)	$7.914x10^{-4}$
**LPC a C26:0**	0.162	(0.030;0.295)	0.017	0.013	(-0.146;0.172)	0.869	0.147	(-0.074;0.368)	0.190
**PC aa C30:0**	0.100	(-0.068;0.268)	0.242	0.012	(-0.228;0.251)	0.922	0.201	(-0.118;0.519)	0.215
**PC aa C32:0**	0.109	(0.013;0.206)	0.026	-0.039	(-0.179;0.102)	0.588	0.076	(-0.167;0.319)	0.536
**PC aa C32:1**	0.352	(0.092;0.612)	0.008	0.112	(-0.149;0.373)	0.397	0.661	(0.021;1.300)	0.043
**PC aa C32:2**	0.251	(0.087;0.414)	0.003	0.033	(-0.168;0.234)	0.748	0.321	(-0.030;0.671)	0.072
**PC aa C34:1**	0.197	(0.097;0.297)	$1.422x10^{-4}$	0.031	(-0.095;0.157)	0.630	0.216	(-0.098;0.529)	0.176
**PC aa C34:2**	0.144	(0.070;0.218)	$1.701x10^{-4}$	-0.046	(-0.146;0.053)	0.355	0.041	(-0.168;0.250)	0.699
**PC aa C34:3**	0.178	(0.024;0.333)	0.024	0.015	(-0.178;0.209)	0.878	0.238	(-0.096;0.573)	0.161
**PC aa C34:4**	0.322	(0.116;0.529)	0.002	0.033	(-0.228;0.295)	0.800	0.436	(0.041;0.831)	0.031
**PC aa C36:3**	0.263	(0.153;0.373)	$5.599x10^{-6}$	-0.014	(-0.152;0.124)	0.839	0.280	(0.037;0.524)	0.024
**PC aa C36:4**	0.295	(0.188;0.401)	$1.887x10^{-7}$	-0.069	(-0.205;0.067)	0.315	0.262	(0.009;0.515)	0.042
**PC aa C36:6**	0.154	(-0.071;0.379)	0.179	0.015	(-0.231;0.262)	0.901	0.477	(0.133;0.820)	0.007
**PC aa C38:6**	0.170	(0.012;0.327)	0.035	-0.095	(-0.251;0.060)	0.229	0.409	(0.203;0.615)	$1.345x10^{-4}$
**PC ae C42:0**	0.115	(0.030;0.200)	0.009	-0.058	(-0.159;0.043)	0.258	0.124	($1.475x10^{-4}$;0.247)	0.050
**SM C20:2**	0.143	(-0.002;0.289)	0.054	-0.010	(-0.178;0.158)	0.906	0.319	(0.066;0.573)	0.014

Analyses adjusted for age, time of blood sampling, fasting time (RCS), waist circumference, white blood cell count, red blood cell count, thrombocytes count, and cystatin C concentrations. OC non-users without menstrual bleeding at the time of assessment used as the reference group (N = 92).

Metabolites presented if the effect of OC use was Bonferroni-corrected significant (171 tests: $p < 2.924x10^{-4}$) in SHIP-TREND.

Table S7. Meta-analytic results for metabolites associated with OC intake in the dependence of menstrual bleeding at the time of assessment.

	Meta-analyses									Heterogeneity					
	no menstruation			menstruation						no menstruation		menstruation			
	OC users (N = 99)			OC non-users (N = 56)			OC users (N = 17)			OC users		OC non-users		OC users	
	**b**	**95%-CI**	**p-value**	**b**	**95%-CI**	**p-value**	**b**	**95%-CI**	**p-value**	**b**	**p-value**	**b**	**p-value**	**b**	**p-value**
**Carnitine**	-0.156	(-0.217;-0.094)	6.786×10^{-7}	0.045	(-0.038;0.128)	0.287	-0.040	(-0.137;0.057)	0.425	17.833	0.270	0.000	0.535	0.000	0.457
**Citrulline**	-0.256	(-0.345;-0.166)	2.426×10^{-8}	0.078	(-0.024;0.179)	0.133	-0.027	(-0.181;0.126)	0.728	72.274	0.058	0.000	0.724	0.000	0.860
**Glutamine**	-0.235	(-0.295;-0.175)	1.453×10^{-14}	0.034	(-0.035;0.103)	0.332	-0.123	(-0.192;-0.053)	5.036×10^{-4}	0.000	0.743	0.000	0.899	0.000	0.370
**Glycine**	-0.497	(-0.604;-0.390)	8.906×10^{-20}	0.048	(-0.076;0.173)	0.447	-0.413	(-0.572;-0.254)	3.585×10^{-7}	0.000	0.352	0.000	0.629	65.781	0.087
**Ornithine**	-0.399	(-0.484;-0.314)	3.179×10^{-20}	0.052	(-0.053;0.158)	0.332	0.011	(-0.198;0.220)	0.919	74.275	0.049	27.896	0.239	66.669	0.083
**Tyrosine**	-0.287	(-0.369;-0.205)	7.777×10^{-12}	0.132	(0.028;0.236)	0.013	0.040	(-0.163;0.242)	0.702	0.000	0.773	0.000	0.531	0.000	0.457
LPC a C14:0	*0.047*	*(3.415×10^{-4};0.095)*	*0.048*	*0.070*	*(0.004;0.136)*	*0.038*	*0.091*	*(-0.007;0.188)*	*0.069*	*94.376*	*2.478×10^{-5}*	*0.000*	*0.436*	*70.795*	*0.064*
**LPC a C17:0**	-0.331	(-0.418;-0.245)	6.046×10^{-14}	0.069	(-0.042;0.180)	0.223	-0.190	(-0.369;-0.012)	0.037	78.164	0.032	66.019	0.086	90.413	0.001
**LPC a C18:0**	-0.423	(-0.505;-0.342)	2.876×10^{-24}	0.116	(0.021;0.210)	0.016	-0.334	(-0.485;-0.184)	1.387×10^{-5}	75.428	0.044	0.000	0.915	89.153	0.002
**LPC a C18:1**	-0.379	(-0.461;-0.298)	7.419×10^{-20}	0.101	(0.003;0.199)	0.043	-0.168	(-0.312;-0.024)	0.023	74.821	0.046	42.244	0.188	91.183	7.577×10^{-4}
**LPC a C18:2**	-0.509	(-0.605;-0.412)	4.382×10^{-25}	0.063	(-0.044;0.170)	0.249	-0.331	(-0.518;-0.145)	4.998×10^{-4}	0.000	0.332	0.000	0.399	92.415	2.825×10^{-4}
**LPC a C20:4**	-0.251	(-0.326;-0.176)	5.374×10^{-11}	0.049	(-0.048;0.145)	0.323	-0.202	(-0.366;-0.038)	0.016	64.930	0.091	0.000	0.408	83.501	0.014
**LPC a C26:0**	0.173	(0.087;0.260)	8.871×10^{-5}	0.016	(-0.080;0.111)	0.747	0.278	(0.143;0.413)	5.288×10^{-5}	0.000	0.827	0.000	0.970	55.113	0.136
**PC aa C30:0**	0.348	(0.242;0.453)	9.789×10^{-11}	0.138	(-0.003;0.279)	0.055	0.280	(0.078;0.481)	0.007	92.924	1.704×10^{-4}	40.316	0.196	0.000	0.525
**PC aa C32:0**	0.157	(0.090;0.224)	4.643×10^{-6}	0.044	(-0.039;0.128)	0.297	0.121	(0.009;0.233)	0.034	47.468	0.168	52.866	0.145	0.000	0.680
**PC aa C32:1**	0.601	(0.452;0.749)	2.503×10^{-15}	0.197	(0.032;0.362)	0.020	0.431	(0.130;0.731)	0.005	81.324	0.021	0.000	0.404	0.000	0.419
**PC aa C32:2**	0.339	(0.230;0.447)	9.435×10^{-10}	0.060	(-0.078;0.197)	0.393	0.291	(0.032;0.550)	0.027	51.125	0.153	0.000	0.714	0.000	0.804
**PC aa C34:1**	0.258	(0.189;0.327)	3.131×10^{-13}	0.080	(-0.006;0.166)	0.070	0.241	(0.056;0.427)	0.011	64.966	0.091	9.510	0.293	0.000	0.842
**PC aa C34:2**	0.198	(0.143;0.254)	2.183×10^{-12}	0.011	(-0.057;0.079)	0.744	0.099	(-0.044;0.242)	0.176	79.982	0.025	60.910	0.110	0.000	0.450
**PC aa C34:3**	0.351	(0.257;0.445)	2.080×10^{-13}	0.146	(0.027;0.264)	0.016	0.206	(0.011;0.400)	0.038	87.204	0.005	65.364	0.089	0.000	0.811

**PC aa C34:4**	0.527	(0.408;0.647)	$5.027x10^{-18}$	0.150	(0.006;0.295)	0.042	0.437	(0.171;0.704)	0.001	82.887	0.016	12.203	0.286	0.000	0.992
**PC aa C36:3**	0.308	(0.236;0.380)	$7.124x10^{-17}$	0.024	(-0.064;0.113)	0.589	0.206	(0.046;0.365)	0.011	13.600	0.282	0.000	0.468	0.000	0.421
**PC aa C36:4**	0.326	(0.252;0.401)	$8.596x10^{-18}$	-0.012	(-0.105;0.081)	0.797	0.191	(0.047;0.334)	0.009	0.000	0.407	23.784	0.252	0.000	0.496
**PC aa C36:6**	0.382	(0.265;0.500)	$1.836x10^{-10}$	0.147	(0.007;0.288)	0.040	0.438	(0.242;0.634)	$1.169x10^{-5}$	82.018	0.018	40.282	0.196	0.000	0.787
**PC aa C38:6**	0.239	(0.146;0.332)	$5.025x10^{-7}$	-0.004	(-0.110;0.101)	0.935	0.357	(0.207;0.507)	$3.026x10^{-6}$	15.924	0.275	59.983	0.114	0.000	0.465
**PC ae C42:0**	0.141	(0.086;0.196)	$5.339x10^{-7}$	-0.032	(-0.105;0.041)	0.385	0.117	(0.033;0.201)	0.006	0.000	0.430	0.000	0.463	0.000	0.886
**SM C20:2**	0.220	(0.119;0.320)	$1.865x10^{-5}$	0.021	(-0.093;0.134)	0.718	0.320	(0.140;0.501)	$5.067x10^{-4}$	52.501	0.147	0.000	0.619	0.000	0.990

Analyses adjusted for age, time of blood sampling, fasting time (RCS), waist circumference, white blood cell count, red blood cell count, thrombocytes count, and cystatin C concentrations. OC non-users without menstrual bleeding at the time of assessment used as the reference group (N = 219).
Metabolites presented if the effect of OC use was Bonferroni-corrected significant (171 tests: $p < 2.924x10^{-4}$) in SHIP-TREND. Italics = meta-analytic effect not significant in any of the three groups.

Table S8. Mediating effects of cortisol in women without menstrual bleeding at the time of assessment.

	SHIP-TREND (N = 190)			SHIP-2 (N = 128)			Meta-analysis						Heterogenity			
	ind. effect	95%-CI	p-value	ind. effect	95%-CI	p-value	ind. effect	95%-CI	p-value	% mediated	95%-CI	p-value	ind. effect	p-value	% mediated	p-value
**Carnitine**	-0.090	(-0.166;-0.015)	0.019	-0.007	(-0.069;0.055)	0.817	-0.041	(-0.089;0.007)	0.095	52.1	(-5.417;109.636)	0.076	63.990	0.096	0.0	0.929
**Citrulline**	-0.025	(-0.125;0.075)	0.624	-0.025	(-0.116;0.066)	0.592	-0.025	(-0.092;0.042)	0.468	7.4	(-23.850;38.567)	0.644	0.000	0.999	0.0	0.977
**Glutamine**	-0.055	(-0.124;0.014)	0.116	0.009	(-0.041;0.059)	0.728	-0.013	(-0.054;0.027)	0.522	7.6	(-12.925;28.215)	0.466	54.107	0.140	39.7	0.198
**Glycine**	-0.195	(-0.320;-0.069)	0.002	-0.065	(-0.190;0.061)	0.311	-0.130	(-0.218;-0.041)	0.004	22.6	(2.091;43.074)	0.031	51.645	0.150	56.0	0.132
**Ornithine**	-0.125	(-0.216;-0.035)	0.007	-0.038	(-0.148;0.072)	0.499	-0.090	(-0.160;-0.020)	0.012	23.4	(4.208;42.553)	0.017	31.041	0.229	0.0	0.584
**Tyrosine**	-0.134	(-0.224;-0.044)	0.004	0.101	(0.002;0.199)	0.045	-0.027	(-0.093;0.040)	0.427	10.8	(-19.811;41.472)	0.488	91.597	$5.610x10^{-4}$	85.9	0.008
**LPC a C17:0**	-0.049	(-0.147;0.049)	0.327	-0.134	(-0.260;-0.008)	0.037	-0.081	(-0.158;-0.004)	0.040	24.1	(0.555;47.641)	0.045	8.506	0.296	0.0	0.746
**LPC a C18:0**	-0.074	(-0.170;0.021)	0.128	-0.100	(-0.201;$5.912x10^{-4}$)	0.051	-0.087	(-0.156;-0.017)	0.014	18.6	(2.409;34.715)	0.024	0.000	0.715	0.0	0.866
**LPC a C18:1**	-0.046	(-0.141;0.048)	0.335	-0.097	(-0.197;0.003)	0.058	-0.070	(-0.139;-0.001)	0.045	17.2	(0.069;34.421)	0.049	0.000	0.473	0.0	0.847
LPC a C18:2	*-0.123*	*(-0.229;-0.017)*	*0.023*	*-0.117*	*(-0.228;-0.006)*	*0.040*	*-0.120*	*(-0.197;-0.043)*	*0.002*	*22.0*	*(6.412;37.590)*	*0.006*	*0.000*	*0.941*	*0.0*	*0.650*
**LPC a C20:4**	0.043	(-0.054;0.140)	0.386	-0.075	(-0.165;0.016)	0.107	-0.020	(-0.086;0.046)	0.557	14.3	(-11.157;39.750)	0.271	66.745	0.083	36.7	0.209

**LPC a C26:0**	0.066	(-0.044;0.175)	0.239	0.092	(-0.001;0.185)	0.053	0.081	(0.010;0.152)	0.025	35.2	(-107.320;177.630)	0.629	0.000	0.723	0.0	0.960
**PC aa C32:0**	0.116	(0.021;0.211)	0.016	0.050	(-0.022;0.123)	0.172	0.075	(0.017;0.132)	0.011	55.0	(-4.934;114.833)	0.072	14.195	0.280	0.0	0.988
PC aa C32:1	*0.139*	*(-0.019;0.296)*	*0.085*	*0.333*	*(0.135;0.531)*	*9.631x10^{-4}*	*0.214*	*(0.091;0.338)*	*6.666x10^{-4}*	*18.9*	*(-4.395;42.185)*	*0.112*	*56.039*	*0.131*	*0.0*	*0.869*
**PC aa C32:2**	0.020	(-0.096;0.135)	0.737	0.128	(0.002;0.253)	0.046	0.069	(-0.016;0.154)	0.111	9.2	(-18.960;37.276)	0.523	34.941	0.215	0.0	0.369
PC aa C34:1	*0.123*	*(0.029;0.216)*	*0.010*	*0.086*	*(0.021;0.150)*	*0.009*	*0.098*	*(0.045;0.151)*	*3.136x10^{-4}*	*40.1*	*(13.387;66.814)*	*0.003*	*0.000*	*0.522*	*0.0*	*0.851*
**PC aa C34:2**	0.100	(0.023;0.177)	0.011	0.043	(-0.009;0.096)	0.104	0.061	(0.018;0.104)	0.006	34.7	(6.933;62.501)	0.014	30.224	0.231	0.0	0.874
PC aa C34:3	*0.131*	*(0.035;0.226)*	*0.007*	*0.133*	*(0.014;0.252)*	*0.029*	*0.131*	*(0.057;0.206)*	*5.357x10^{-4}*	*28.4*	*(4.843;51.991)*	*0.018*	*0.000*	*0.979*	*0.0*	*0.924*
PC aa C34:4	*0.200*	*(0.062;0.337)*	*0.004*	*0.175*	*(0.028;0.322)*	*0.020*	*0.188*	*(0.088;0.289)*	*2.466x10^{-4}*	*30.7*	*(7.620;53.853)*	*0.009*	*0.000*	*0.812*	*0.0*	*0.913*
PC aa C36:3	*0.172*	*(0.081;0.262)*	*2.032x10^{-4}*	*0.110*	*(0.037;0.182)*	*0.003*	*0.134*	*(0.077;0.190)*	*3.405x10^{-6}*	*45.1*	*(22.856;67.394)*	*7.137x10^{-5}*	*9.413*	*0.293*	*0.0*	*0.694*
PC aa C36:4	*0.208*	*(0.099;0.316)*	*1.706x10^{-4}*	*0.101*	*(0.033;0.170)*	*0.004*	*0.132*	*(0.074;0.189)*	*7.876x10^{-6}*	*41.3*	*(21.771;60.843)*	*3.412x10^{-5}*	*62.235*	*0.104*	*21.1*	*0.260*
**PC aa C38:6**	0.108	(0.003;0.214)	0.044	0.098	(-0.014;0.210)	0.086	0.104	(0.027;0.180)	0.008	37.8	(-4.804;80.332)	0.082	0.000	0.899	0.0	0.979
PC ae C42:0	*0.124*	*(0.059;0.189)*	*1.850x10^{-4}*	*0.057*	*(0.009;0.105)*	*0.020*	*0.081*	*(0.042;0.119)*	*4.226x10^{-5}*	*70.8*	*(16.779;124.910)*	*0.010*	*62.361*	*0.103*	*0.0*	*0.723*
**SM C20:2**	0.109	(3.617x10^{-4};0.218)	0.049	0.124	(0.011;0.237)	0.031	0.117	(0.038;0.195)	0.004	36.1	(-6.289;78.519)	0.095	0.000	0.853	0.0	0.951

Analyses adjusted for age, time of blood sampling, fasting time (RCS), waist circumference, white blood cell count, red blood cell count, thrombocytes count, and cystatin C concentrations.

Metabolites presented if the effect of OC use was Bonferroni-corrected significant (171 tests: $p < 2.924x10^{-4}$) in SHIP-TREND. LPC a C14:0, PC aa C30.0, and PC aa C36:6 missing due to non-significant associations between these metabolite concentrations and cortisol in SHIP-2. Italics = significant indirect effect in meta-analysis after Bonferroni-correction for mediation analyses (24 tests. $p < 0.002$)
